# Mental Representation of Word Family Structure: The Case of German Infinitives, Conversion Nouns and Other Morphologically Related Forms

**DOI:** 10.3389/fpsyg.2022.910849

**Published:** 2022-07-27

**Authors:** Andreas Opitz, Denisa Bordag, Alberto Furgoni

**Affiliations:** ^1^Herder-Institut, Leipzig University, Leipzig, Germany; ^2^University of Haifa, Haifa, Israel; ^3^BCBL-Basque Center on Cognition, Brain and Language, Donostia-San Sebastian, Spain

**Keywords:** mental lexicon, priming, word family, morphology, conversion nouns, infinitives, L1, L2

## Abstract

This study investigates how two non-finite forms, infinitives and conversion nouns, are represented in the mind of L1 and L2 speakers and what is their relationship to other members of the corresponding word family. German native speakers and proficient German learners with Czech as L1 participated in four overt priming experiments involving a grammatical judgement task. We investigated the relationship between infinitives (Experiment 1) and conversion nouns (Experiment 2) and formally identical verbal or noun forms. We further focussed on the relationship between conversion nouns and regular nominal derivation forms with two derivational suffixes: *-er* and *-ung* (Experiments 3 and 4). Our results show that the two non-finite forms differ in their relations to other members of a word family and do not constitute a special class of non-finites as suggested in previous literature. While German infinitives seem to be closer related to finite verbal forms, conversion nouns behave in the same way as other regular nominal derivatives within the same word family. As for the German L1 and L2 contrast, no significant difference in the mental representation of the examined forms was found. This finding suggests that with respect to the explored phenomena, proficient learners rely on the same linguistic organisation as L1 speakers.

## Introduction

A word family comprises morphologically related words that share the same root. Previous research has shown that morphologically related word forms typically expedite each other’s recognition in priming, indicating that their representations are not independent (see, e.g., McQueen and Cutler, 1998 and Schmidtke and Kuperman, 2020 for overviews of evidence from morphological priming). Surprisingly, relatively little research directly addresses the question of word family structure. Nevertheless, some topics that can be subsumed under it have been explored rather extensively both in L1 and L2, such as the research on inflection and derivation (see Silva and Clahsen, 2008 or Jacob et al., 2018 for overviews). This line of research suggests that the degree to which word family members affect each other depends on the type of morphological relation between them. Although these studies do not yield completely homogeneous results, the general trend indicates that inflectionally related members of word families are more closely tied and possibly share the same lexical entry at least in L1. Conversely, derivationally related members have a looser relation. Overall, the fact that facilitation in priming studies is not equally strong between the word family members implies that a word family is not a simple list, but rather has a structure which we want to explore in the current study (see van de Vijver and Baer-Henney, 2019). In particular, we focus on the position of two non-finite forms, infinitives and conversion nouns, in the word family and on the relationship between them. These forms can be seen as borderline phenomena in several respects (see below) and have been rarely addressed in psycholinguistic research on morphological relationships between related word forms. Exploration of their status in L1 and L2 families could shed more light on differences and similarities between native and non-native representation and processing.

Traditionally, non-finite verbal forms are those deverbal forms that are not inflected for tense and lack subject agreement (Chamoreau and Estrada-Fernández, 2016). According to some typological approaches, infinitives and conversion nouns (as well as participles and converbs) belong to the class of so-called non-finites that exists in addition to the lexical classes of nouns and verbs (see Ylikoski, 2003 for an overview). (Non)finiteness and nominalisation are generally described as correlated and interacting (Bisang, 2001; Givón, 2001) and infinitives and conversion nouns are sometimes classified as action nominals (Nikolaeva, 2010). At the same time, differences within the class of non-finites have also been pointed out. As an example, Ylikoski (2003) mentions that while conversion nouns can be best defined as verbal nouns and participles as verbal adjectives (word class approach), infinitives and converbs are better defined through reference to their complementary functions (infinitives being obligatory in a sentence while converbs optional; functional approach).

In German, all infinitives consist of the stem and typically the ending -*en* (occasionally -*eln* and -*ern*. E.g. *miet*-*en* ‘rent’). Similar to English, there are two forms of an infinitive, a bare infinitive (*mieten*) and a *zu-*infinitive (*zu mieten*) that approximately corresponds to the to-infinitive in English. As in other languages, German infinitives have complementary syntactic functions. There is no controversy in traditional grammars that infinitives are verbal forms, and they appear as citation forms for the whole verbal paradigm in dictionaries. Infinitives are also used as the representative forms in L2 German teaching and learning and are thus learnt during the first stages of L2 acquisition. In our study, we compare native German speakers and advanced German learners at B2/C1 level with L1 Czech. Czech infinitives of all conjugational classes have a non-homonymous ending -*t* that is usually preceded by a conjugation class stem suffix, for example *zpív*-*a*-*t* ‘to sing’ or *prac*-*ova*-*t* ‘to work’. While infinitives in German formally overlap with other verbal and nominal forms, Czech infinitives are non-homonymous. This implies a different formal relationship between them and other word family members compared to German and makes transfer of abstract word family structure (features) undesirable for achieving native-like representation.

Conversion or zero-derived nouns can be formed from all German verbs in an entirely regular and productive manner. Any infinitive form can be converted into a conversion noun, for example *mieten* ‘to rent’ and *das Mieten* ‘the renting’. These forms are semantically transparent in that their meaning can be regularly derived from their base (abstraction of the action corresponding to the English gerund). For this reason, the classification of conversion forms as inflected or derived is controversial. In general, derivation potentially implies a word class change whereas inflection does not (Haspelmath, 1996). Conversion, however, can be seen as a process that is ‘inflectional in the sense that it is regular, general and productive, but nonetheless transpositional’ (Bauer, 2004). Haspelmath (1996) consequently suggested an extension of the definition of inflection, subsuming also the so-called transpositional and word-class-changing inflective forms that include conversion. Despite the properties that bind conversion nouns with other non-finite verbal forms (cf. the term action nominals in typology), they are traditionally considered derived nouns in German grammars as they function as heads of NPs with similar functions to NPs with underived nouns as their heads. All such conversion nouns have the same grammatical properties: they are all uncountable, have a neutral gender and so-called strong declension. In Czech, action nominals that correspond in their function to German conversion nouns are not homonymous with either infinitives or any other inflected verb form. They are derived from past participles by the suffix -*í* (e.g., *zpí*-*á*-*n*-*í* ‘singing’, *prac*-*ová*-*n*-*í* ‘working’). However, the concept of conversion is familiar to Czech native speakers as it exists, for example between adjectives and nouns. The explored type of conversion in German is structurally very simple, completely regular and very productive. It thus enables L2 learners to enlarge their competence significantly at very low costs. As such, conversion is typically learnt and mastered rather early in L2 German; at the latest at B1 level (at least for Czech learners). However, given the language specific characteristics of conversion nouns in Czech and German, the formal relation between them and infinitives (and other related forms) cannot be simply transferred, if native-like representation of word family structure is to be achieved in L2 German.

Previous research indicated that processing and representation of morphologically complex words may be language or language-type specific and that fundamental cross-linguistic differences may lie behind different results and interpretations (e.g., Li et al., 2006; Verhoeven and Perfetti, 2017; Schmidtke and Kuperman, 2019; or Leminen et al., 2019). As an example, the results of a priming study by Smolka et al. (2015) show that in more synthetic German, derived members of word families are more closely tied *via* their stems than is the case in more analytic English. Both experimental evidence (see Smolka and Ravid, 2019 and Milin et al., 2018 for overview) and computational models (Günther et al., 2019) indicate that quantitatively characterised differences between languages (e.g., degree of morphological analysis vs. synthesis) may result in behavioural differences in morphological representation and processing.

Another factor that has turned out significant for the exploration of relationships between morphologically related words in particular with respect to L1 vs. L2 differences is priming direction. Early priming studies (e.g., Feldman and Fowler, 1987; Schriefers et al., 1992) revealed that directionality matters and that different results may be obtained for the same word forms when primes and targets are switched. Most psycholinguistic research that reports asymmetric priming investigated the processing of inflected adjectives, or stem alternatives in irregular verb morphology in German. Clahsen et al. (2001) for instance, report results of a cross-modal priming study that revealed priming asymmetries between different inflected forms of adjectives. Adjectives with -*s* affix (*klein*-[əs]) as primes facilitated the recognition of targets with -*e* affixation (*klein*-[ə]) as effectively as the corresponding identity controls, but priming for the reversed order led to less facilitation. These asymmetries were explained through differently complex morpho-syntactic feature specifications of the investigated forms with more specific forms facilitating recognition of less specific forms better than vice versa. Asymmetric priming was also reported for stem allomorphy in irregular verbs (e.g., English: throw vs. threw [+Past], German: *werf*- vs. *warf*- [+Past]; e.g., Krause et al., 2015). The results again showed that when primes and targets were in a more specified-to-less specified (*warf*- [+Past] → *werf*-) direction, stronger priming (near repetition) was observed than for the reverse order (i.e., *werf*- → *warf*- [+Past]).

Interestingly, similar studies that also included groups of advanced non-native L2 learners (Krause et al., 2015; Bosch and Clahsen, 2016; Bosch et al., 2017, 2019) obtained such asymmetric priming in L1, but at the same time report clear native-nonnative differences, with asymmetric priming either absent in L2 (Bosch and Clahsen, 2016) or even with a tendency of a reversed direction (Krause et al., 2015). The authors argue for a stronger reliance of L2 learners on storage and retrieval of whole word form representations (more lexicon-based processing) then on decoding of morphological structure of complex words (rule-based, hierarchical processing; see also Neubauer and Clahsen, 2009). As illustrated, bidirectional priming relationships between word forms can shed light on the structure of morphological word families and, in particular, on L1 vs. L2 differences, and, therefore, we included this in the design of our experiments.

As mentioned, the structure of German word families has been rarely directly addressed in previous research. An exception is a study with auditory lexical decision experiments by van de Vijver and Baer-Henney (2019). The authors explored relationships among inflected nouns (singular and plural) and derived nouns (diminutives). Based on their results, they propose a frame representation of word family structure, in which properties of a central node are represented as attribute-value structures (with attributes being functions that return a value; cf. Crysmann and Bonami, 2016). Inflectional classes of German nouns are thus represented as frames, in which the central node of each class is a category noun, and the attributes and their values are the morphological and phonological properties that define an inflectional class. In the case of diminutives, the change in their meaning relative to the central node is analysed as a shift of the referent from one node to another (Andreou, 2018). Within one word family, inflectionally related words from one paradigm and the derived words are represented in one frame (cf. Figure 5 in their paper), but the inflected forms are more strongly associated with each other than with the derived forms, which have a different referential node. Even though the approach is appealing, it addresses only a limited fraction of morphological phenomena and is thus unsuitable for assessing our data. For example singular is taken as a default number and plural is an attribute modelled the same way as case (e.g., ‘has nominative’, ‘has genitive’, ‘has plural’). However, German nouns do not need to have only one plural form as suggested by their Figure 1 representing the frame of the word *Tag*, but leaving out the dative plural form *Tagen*. In addition, syncretism is not modelled and so it is unclear how dative and accusative forms that are homonymous with the nominative form in singular would be represented. Modelling of verbal paradigms with significantly more features than nouns also seems difficult to imagine within the given frame approach. Clearly, more data are needed to reach the state in which it is possible to successfully model the morphological family structure in its completeness. With the present study, we want to address to this issue.

**Figure 1:**
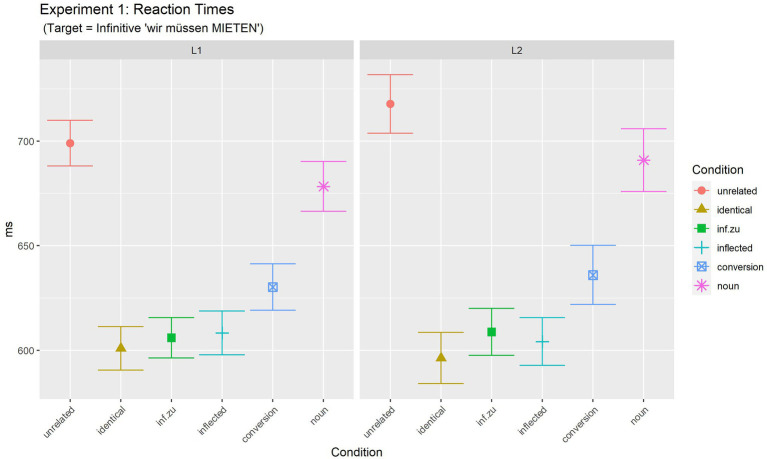
Experiment 1 (target: infinitive): mean reaction times for target phrases.

To our knowledge, with the exception of several studies by Bordag et al. (Bordag and Opitz, 2021, 2022; Opitz and Bordag, 2021), there is no research in German that would systematically explore the relation between infinitives and conversion nouns or between them and other morphologically related words. In particular, conversion nouns have not been employed in previous research. Infinitive forms have been included in some priming studies (e.g., Jacob et al., 2018 on L1 and L2 German); they were however used in isolation, which undermines their actual syntactic function in the experiments. In particular, since the verb forms with an -*en* ending are homonymous and a form like *mieten* can function as an infinitive, but in appropriate contexts also as the 1st or 3rd person plural (*wir mieten*, *sie mieten*) or—as already demonstrated—also as a conversion noun or, for example as a dative plural form of the derivation *die Miete* ‘the rent’ (e.g., *mit den Mieten* ‘with the rents’). When homonymous forms are a subject of a priming experiment that addresses their various functions, it is indispensable that they appear in syntactically unambiguous contexts that determine their function. Consequently, only unmasked priming is suitable for such purposes.^1^ Moreover, an advantage of such a paradigm is that it provides a more ecologically valid way of examining word recognition than single word recognition paradigms.

Such an approach was also employed in Bordag and Opitz (2021) and Opitz and Bordag (2021) in several visual priming experiments that, among other things, explored the relation of infinitives and conversion nouns with homonymous inflected forms. The authors compared priming between overtly identical primes and targets while manipulating the function of the primes. The primes and targets were always introduced by a brief context presented in a separate step preceding the prime or target word presentation. The target was always the inflected form *wir*—*MIETEN* ‘we rent’ and the primes formed the following conditions: conversion: *das*—*MIETEN* ‘the renting’; infinitive: *wir müssen*—*MIETEN* ‘we have to rent’; inflected: *sie*—*MIETEN* ‘they rent’; countable derived noun: *mit den zwei*—*MIETEN* ‘with the two rents’; identical: *wir*—*MIETEN* and unrelated: *wir*—*WEINEN* ‘we cry’. The results showed that full priming was observed in the identical and inflected conditions, partial priming in the infinitive and conversion condition and no priming in the countable derived noun condition. The countable nouns are unproductive derivations from the verb base, whose semantics (in relation to the base) are unpredictable. As an example, while *MIETE* means ‘a rent’ (derived from the verb *mieten*), *FLIEGE* means ‘a fly’ (the insect), derived from the verb *fliegen* ‘to fly’. Employment of overtly identical forms^2^ with different functions eliminated possible effects of phonological overlap and surface frequency effects (see Schriefers et al., 1992).

It has been argued that these properties rather than morphological relations might be responsible for differences in the degree of facilitation in priming. Clearly, when identical forms are used, such arguments are irrelevant, and potential priming differences between them can only be attributed to their different functions. As mentioned, Bordag and colleagues report partial priming for both infinitives and conversion nouns as primes for inflected verbs as targets. This contrasts with full priming that was observed between inflected forms, and with no priming between the derived countable nouns and the finite target form. The authors argue that the partial priming indicates that infinitives and conversion nouns could fall in a shared category of non-finites. Members of this category would be in a different relation to other members of the given word family, such as inflected forms and other derived nouns. Interestingly, exactly the same priming patterns were obtained for both German native speakers and advanced L2 German learners with L1 Czech, indicating that the explored members of the given word families were in the same relationships to each other both in L1 and in L2. However, what these studies did not test was the directionality of the priming, namely when the two critical forms would be in the position of targets. Moreover, the countable derived nouns are unproductive derivations which contrasts with conversion nouns that are highly productive and regular. It thus remains open, how, in particular, conversion nouns relate to other nouns within the word family which are also productively derived and semantically regular/transparent. Both questions are addressed in the current study.

## The Present Study

In this study, we aim at understanding in which psycholinguistic relation German infinitives and conversion nouns are to each other and to other members of their word family. In particular, we are interested in their relation to finite forms and derived non-conversion nouns. If, within a given word family, infinitives and conversion nouns were in the same relationship to the members of the corresponding verb paradigm and this relationship would at the same time differ from the relationships between the inflected verb forms, on the one hand, and other derived nouns, on the other, it would support the psycholinguistic existence of a separate (and internally homogenous) class of non-finites, as suggested by some typological approaches (see Ylikoski, 2003 for an overview). These relations will be investigated based on the priming directionality of the different forms. We explore the topic also from the perspective of L2 acquisition and compare the L1 and L2 word family structures, in particular the position of infinitives and conversion nouns in them given the cross-linguistic differences between their forms in German and Czech.

This study is composed of four main experiments. We also briefly present the results of two other experiments: Supplementary Experiment S5 further corroborates what was found in the previous experiments. An additional control experiment (Supplementary Experiment S6) systematically addresses two subgroups of stimuli that appear in the main experiments (details concerning the two additional experiments can be found in the Supplementary Material). Concerning the four central experiments, each was performed with two populations: adult native speakers of German and advanced German learners at the B2/C1 level with L1 Czech. The two additional experiments were performed only in L1 German. For ease of reading, we state in advance that the pattern of results was the same for L1 and L2: No differences could be observed between the two populations apart from the standard main effect of language indicating that the L2 response times were in general slower than those of the L1 participants. The results of the L2 experiments thus not only contribute to the discussions on differences and similarities between L1 and L2 morphological processing, but—in this particular case—can be also seen as a replication of the L1 results with a different population, thus boosting the validity of the observed patterns.

In Experiments 1 and 2, we explored the (a) symmetry of the relations between infinitives and conversion nouns and other members of their corresponding word families and employed only overtly identical forms both as primes and targets. In Experiments 3 and 4, we included other productively derived nouns in addition to conversion nouns in the paradigm to explore the relation between them. As a result, the overt forms of primes and targets varied.

## Experiments 1 and 2

In Experiments 1 and 2, we explored the assumed category of non-finites with respect to relations between infinitives and conversion nouns and other members of the corresponding word families. In Experiment 1, we employ infinitives as targets and in Experiment 2 conversion nouns. In their analogical experiments, Bordag and Opitz (2021) employed formally identical forms (*MIETEN*) with different morphosyntactic functions (inflected verb form, conversion noun, infinitive and derived countable noun) as primes while the formally identical inflected form (*wir MIETEN*—‘we RENT’) served as a target. Both in L1 and L2, they observed that while inflected verb forms as primes lead to full priming and derived countable nouns to no priming, conversion nouns and infinitives manifested partial priming of the same size. The converging results supported the idea of conversion nouns and infinitives being represented in the same way and possibly forming a category of non-finites that resides between inflection/verbs and derivation/nouns. The current experiments thus add the aspect of priming direction, which enables a finer-grained look at the structure of word families’ representations. We will first report the methods and results of the two experiments and then discuss them in a joint discussion.

### Experiment 1

#### Methods

In the following, we report the methods of the first experiment in detail. Because all reported experiments in this paper were very similar with respect to procedure, we only highlight differences to the first experiment in methods (in particular, materials and participants) for all experiments subsequently reported.

##### Participants

###### L1

All 72 participants were German native speakers (mean age = 28.0, SD = 7.9, range: [19, 57]; sex: 76.4% females, 23.6% males) and most were students of different faculties at Leipzig University.

###### L2

A total of 64 non-native participants entered the final analysis. All were native Czech speakers (mean age = 24.6, SD = 4.7, range: [19, 44]; sex: 70.3% females, 29.7% males). They were all students at Charles University in Prague. Language proficiency of all non-native participants was assessed prior to the experimental session using three different measures: a shortened version of the Goethe Test, an online version of DiaLang and a self-evaluation of each participant using a language-skill questionnaire. Only participants that reached levels B2 and C1 according to the Common European Framework of Reference for Languages (CEFR) at the three language assessments were tested in the experiment (most participants reached both B2 and C1 depending on the test).

##### Materials

Twenty-four German verbs with corresponding homophonous nouns representing countable objects [e.g., *mieten* ‘to rent’ (verb) and *Mieten* ‘rents’ (noun, pl.)] were selected.^3^ The stimuli were carefully controlled so that the frequency of both verbs and their countable noun counterparts was average or high (ranged between frequency class 7 and 17 according to Leipzig Wortschatz Projekt^4^). Moreover, care was taken that the words were well known to L2 learners at the B2 level by means of a pretest. Given that the number of German verbs with corresponding homophonous nouns is rather limited, the relative frequencies of both verbal and nominal forms could not be perfectly matched. Furthermore, the plural forms of half of the homophone countable nouns are formed by affixation in -*n* (*Miete-n* ‘the rents’), while the rest by affixation in -*e* (*Spiele-e* ‘the games’).^5^ However, all nouns in the critical countable noun condition (see below) were presented in dative plural context and thus all ended in -*en*: they either exhibit their regular *n*-plural marker throughout all cases [*mit den Miete-n* ‘with the rents(dative)’], or an additional marker *-n* is added to their regular plural marker in dative contexts (*mit den Spiel-e-n* ‘with the games’). Moreover, the type of relation between a verb and a countable noun is not homogeneous. Although it is notoriously difficult to determine the direction of derivation with certainty, in some cases the verb is most probably derived from the noun historically (der *Löffel* ‘the spoon’—*löffeln* ‘to eat with a spoon’), while in other cases, the noun is most probably derived from the verb (*das Spiel* ‘the game’—*spielen* ‘to play’). In a control experiment that targeted this particular question and that employed the same experimental method, we did not, however, find any evidence that differences in this type of relation would influence participants’ reactions (for details, see Supplementary Experiment S6).

All target verbs were embedded in short phrases that unambiguously marked their syntactic function. The phrases consisted of two parts: first, the pronoun *wir* ‘we’ + the modal verb *müssen* ‘have to’ and second, the critical verb in the infinitive form (e.g., *MIETEN*). The target verb form was always presented in the upper case.^6^ Each target phrase was matched with a prime phrase that also consisted of two parts. The second part was always the homophone form that was formally identical to the target verb form and also written in upper case. It was also preceded by a corresponding first part which determined its syntactic function. In this way, six different priming conditions were created which differed with respect to the relationship of the prime and target verb/noun forms (see Table 1 for an overview).

**Table 1:** Experiment 1: experimental conditions and examples of prime and target phrases.

	Condition	Prime phrase		Target phrase	
		Part 1	Part 2	Part 1	Part 2
1	Identical	wir müssen ‘we have to’	MIETEN ‘rent’	wir müssen ‘we have to’	MIETEN ‘rent’
2	Infinitive.zu	um zu ‘(in order) to’	MIETEN ‘rent’	wir müssen ‘we have to’	MIETEN ‘rent’
3	Inflected	wir ‘we’	MIETEN ‘rent’	wir müssen ‘we have to’	MIETEN ‘rent’
4	Conversion	das ‘the’	MIETEN ‘renting”	wir müssen ‘we have to’	MIETEN ‘rent’
5	Countable noun	mit den zwei ‘with the two’	MIETEN ‘rents’	wir müssen ‘we have to’	MIETEN ‘rent’
6	Unrelated	wir wollen ‘we want to’	WEINEN ‘cry’	wir müssen ‘we have to’	MIETEN ‘rent’

1. **Identical condition**: The prime and the target verb form were both preceded by the pronoun *wir* ‘we’ and the modal verb *müssen* ‘have to’, i.e., the critical word was determined as infinitive. This condition was included to measure the maximal facilitation since the target was fully activated by the prime (full repetition priming).
2. **Infinitive.zu condition:** The syntactic context provided in this condition was the infinitive construction *um zu* ‘in order to’. This condition was included to test whether full repetition of function in prime and target (i.e., infinitive reading) was dependent on the exact repetition of the preceding phrasal contexts (cf. condition 1)—or whether indeed the more abstract grammatical function was primed in the identical condition.
3. **Inflected condition**: The prime form was an inflected verb form, determined by the corresponding plural pronoun *wir* ‘we’ that preceded the verb.
4. **Conversion condition**: The prime form was a converted noun derived from the corresponding target verb. Its syntactic function (i.e., NP) was determined by the preceding neutral definite article *das* ‘the’.
5. **Countable noun condition**: The prime contained an inflected countable concrete noun which was formally identical to the target verb form. The first part of the prime phrase was *mit den zwei* ‘with the two’, which determined that the following noun had to be in dative plural.
6. **Unrelated condition**: Both the target and the prime verb were marked as infinitive forms (as determined by the first person plural pronoun *wir* ‘we’ + the modal *müssen* ‘have to’). Contrary to all other conditions, the verb in the prime phrase (e.g., WEINEN, ‘cry’) was different to the verb in the target phrase (e.g., MIETEN, ‘rent’), but matched for length and frequency.

The 24 critical items were distributed over six different lists with individual per-list randomisation. Each of the six experimental lists consisted of 608 single judgement tasks (24 prime + 24 target phrases of critical trials, 160 × 2 paired filler phrases and 240 single filler phrases). Each item appeared in each list only once in one of the six conditions. The six conditions were counterbalanced across the lists according to Latin Square design and thus represented equally often by four different items on each list. The choice of design—despite each participant encountering only four items per condition (and its possible implications for the statistical power)—was based on the following: (i) the number of suitable items that show all desired characteristics, matched on frequency and length, and that are also familiar to L2 learners is quite limited, (ii) the inclusion of all six conditions allows for a direct between-condition comparison avoiding multiple experimental sessions and the related between-experiment comparisons and (iii) a repeated measure design (i.e., multiple presentation of one item for each participant) might lead to priming effects between different versions of one item and affect the priming sizes and patterns. All 24 critical items consisted of pairs (prime and target) of grammatically correct phrases, except for the unrelated condition. A large number of filler phrases were created and included in each list of the experiment in order to balance the number of nouns and verbs, the use of pronouns, as well as the number of grammatical and ungrammatical forms, and their distributional probabilities within the experiment. The filler items included 80 pairs of prime-target phrases with identical (i.e., repeated) verb forms as second parts of the prime and target phrases. These filler items were similar to the experimental items, but varied regarding the grammaticality of their first and/or second part and the pronouns used (e.g., *sie FEHLEN*—*wir müssen FEHLST* ‘they are absent—we have to be *absent’, with the target being ungrammatical due to incorrect inflection for second person singular). Additionally, 80 pairs of prime-target phrases comprising nouns were used as fillers. They, too, were balanced with respect to the grammaticality of their first and second part. In addition, 240 single, non-paired filler phrases were included (134 verbs, 106 nouns). Ungrammaticality of fillers was achieved by incorrect number and/or person agreement between pronoun and verb form (e.g., *wir LIEST*—‘we reads’), or incorrect number and/or gender agreement between nouns and preceding articles or adjectives [e.g., *das große KATZE—‘the(neuter) big cat(feminine)’].

##### Procedure

Participants gave their informed consent before taking part in the experiment and were tested individually and received a small monetary reward for their participation. During the experiment, they performed a grammaticality judgment task and were instructed accordingly, emphasising that they should respond as fast and accurately as possible. All stimuli were presented visually, using the E-Prime 2.0 software (Schneider et al., 2002). Each trial started with a fixation sign (*) presented at the centre of the screen for 500 ms. Then, a phrase was displayed in two stages. In the first stage, the first part of the phrase, i.e., all material preceding the verb or noun, was presented centred on the screen in black letters on white background (e.g., *wir müssen* ‘we have to’). After 750 ms, these words disappeared and the second part of the phrase (e.g., *MIETEN* ‘rent’) was presented in capital letters, printed in dark green in the same, centred position. Participants were instructed to judge whether the second, green-printed part was a grammatical or ungrammatical completion of the phrase by pressing one of two corresponding buttons. The primary purpose of the judgement task was to assure that participants really read and processed the stimuli. After the participant’s response was registered or after a maximum duration of 2,000 ms, the word disappeared from the screen. Before the next trial started, a blank screen was presented for 600 ms. There were three pauses during the experiment at equidistant intervals. At the beginning of the experiment, there was a short training block consisting of eight trials to familiarise participants with the task. An average experimental session lasted about 35 (L1) and 45 (L2) min. The participants received no feedback during the experiment.

Each participant was administered to one of the six experimental lists. The order of items in the lists was pseudorandomised^7^ for each participant with the following restrictions: No more than five successive trials with the same grammatical status of the phrase (grammatical/ungrammatical) or the same grammatical class of the second part of the phrase (noun/verb) were allowed.

Additionally, there were a minimum of eight intervening filler trials between critical trials, and at least the first three trials after each of the pauses were filler trials.

##### Data Analysis

For all statistical analyses reported in this paper, the statistics software R, version 4.1.2 (R Core Team, 2021) was used. The data were analysed with mixed-effects regression modelling (Baayen et al., 2008) using the R packages *lme4* (Bates et al., 2015) and *lmerTest* (Kuznetsova et al., 2017). A maximal model structure was pursued including items (verb stems) and participants as random effects (cf. Barr et al., 2013). The R package *buildmer* (Voeten, 2022) was used to identify the maximal model structure that was still capable of converging. Starting with an empty random effects structure, terms were added stepwise to the model until convergence of the model could no longer be achieved. The adding of terms was ordered based on the significance of the change in log-likelihood. The result of this procedure was considered the final (i.e., the maximal feasible) model. Final model structures are reported for each analysis below. However, none of these final converging models included the critical predictor (i.e., Condition) as a random slope.^8^ For the final models, significance of fixed effects was evaluated using the R package *lmerTest* (Kuznetsova et al., 2017) with Satterthwaite approximation for degrees of freedom. *Post-hoc* comparisons between multiple conditions were conducted based on the final model’s estimated means using the R package *emmeans* (Lenth, 2022) with Tukey’s adjustment of values of *p* for multiple comparisons.

Raw data of reaction times were first checked for outliers and winsorised with the 2.5 and 97.5 percentile as boundaries: For each participant, all data points that fell below the 2.5th percentile or above the 97.5th percentile were set to these boundary values instead of excluding them from the analysis (cf. Nicklin and Plonsky, 2020). Winsorisation affected 6.2% of data points. In addition, reaction times were log-transformed in order to compensate for non-normality. Data of the L1 and L2 participants were pooled to compare the two tested populations. Accordingly, all statistical analyses comprise a factor Language referring to the two groups of participants, i.e., whether they were native speakers of German (L1) or language learners (L2).

The main analyses comprise reaction times at the target verb forms, which were always embedded in the same syntactic context (*wir müssen* ‘we have to’) and therefore equally easy to process in all conditions. Any differences regarding reaction times at the targets can thus be unambiguously attributed to the influence of the preceding prime phrases.

##### Data Availability

All raw data and the R markdown scripts that produced the reported results are publicly available at the Open Science Framework (OSF): DOI 10.17605/OSF.IO/4FQ8K.

#### Results

##### Accuracy

Target phrases were analysed only if the corresponding prime phrase was judged correctly (exclusion of 264 data points out of 3,264 data points, i.e., 7.5%). The overall accuracy rate for the remaining targets was very high (L1 = 99.1%; L2 = 98.8%), which is why meaningful interpretation of differences between target accuracies is not possible. However, they indicate that L1 and L2 participants were fully capable of dealing with the experimental task. As this observation holds for all reported experiments, we do not report or discuss accuracy scores in detail, but focus on the results of reaction time data.

##### Reaction Times

Analyses were performed over the responses to which participants responded correctly both for the prime and target phrases, i.e., for the analyses of targets, we also pairwise excluded trials with incorrect responses to primes. This led to the exclusion of 8.6% (282 out of 3,264 total trials) of the data. Table 2 summarises mean latencies of the judgement task together with the number of observations for each cell (see also Figure 1).

**Table 2:** Experiment 1: mean reaction times for target phrases in ms, standard deviations (in brackets).

		Unrelated	Identical	Inf.zu	Inflected	Conversion	Noun	*Mean*
L1	RT (SD)	699.0 (180.9)	601.0 (174.4)	606.0 (160.4)	608.3 (175.9)	630.3 (172.2)	678.3 (191.5)	*637.2*
L2	RT (SD)	717.7 (218.4)	596.4 (190.9)	608.8 (175.6)	604.2 (179.1)	636.0 (176.8)	690.9 (225.4)	*642.3*
*Mean*		***708.4***	***598.7***	***607.4***	***606.3***	***633.25***	***684.6***	***639.7***

Statistical analyses [final model: log(RT) ~ Condition*Language + (1 | Participant) + (1 + Language | Item)]^9^ revealed a significant effect of Condition [*F*(5, 2784.8) = 59.32, *p <* 0.001], but no effect of Language [*F*(1, 138.2) = 0.01, *p =* 0.906], nor any interaction [*F*(5, 2784.9) = 0.25, *p =* 0.934].

In order to further investigate the main effect of Prime Condition, differences between the levels of this factor were analysed by computing multiple comparisons of estimated means with adjusted values of *p* (Tukey). Results are summarised in Supplementary Material S1. Results of these comparisons reveal that, overall, only three conditions did not differ from each other. This group consisted of the three fastest conditions: identical (598.8 ms), infinitive.zu (607.3 ms) and the inflected (606.4 ms) condition (all *p* > =0.540 within these three conditions). Thus, full priming (i.e., priming of the same size as full-repetition priming) was observed for both instances of infinitives as primes as well as for inflected verbs in primes. In contrast, for two conditions, partial priming was observed. Both the countable noun condition and the conversion noun condition were significantly faster than the unrelated condition (all *p* ≤ 0.021), but slower than the identical condition (all *p* < 0.001). However, also both partial priming conditions differed from each other: larger (partial) priming was observed for the conversion condition (632.5 ms) than for the countable noun condition (684.2 ms, difference, 51.7 ms, *p* < 0.001). Results are discussed together with the results of Experiment 2 below.

### Experiment 2

#### Methods

Methods were in large parts identical to those of the first experiment. The crucial difference was that the critical word in the target phrases was always presented as a conversion noun. In the following, we only highlight differences to Experiment 1.

##### Participants

###### L1

All 72 participants were German native speakers (mean age = 27.9, SD = 6.1, range: [19, 47]; sex: 56.9% females, 43.1% males), and most of them were students of different faculties at [blinded] University.

###### L2

A total of 59 non-native participants entered the final analysis. All were native Czech speakers (mean age = 25.5, SD = 6.1, range: [19, 44]; sex: 67.8% females, 32.2% males) and were from the same population pool as the participants in the previous experiment. Their L2 skills were also on B2/C1 level and were assessed in the same manner as in Experiment 1.

None of the participants participated in any other experiment of this study.

##### Materials

The same 24 German verbs were used as in Experiment 1. All target forms were unambiguously marked as a conversion noun. Six priming conditions were created which differed with respect to the relationship of the prime and target verb/noun forms (see Table 3 for an overview). In the identical condition, the prime and the target verb form were both preceded by the definite article *das* (‘the’), i.e., the critical word was determined as a conversion noun (‘the renting’). In the ‘conversion.2’ condition, a second type of context was used that also implied conversion noun reading for the critical word (i.e., *beim*—‘during/at the’). This condition was included to test whether the nominative conversion form is fully primed by its dative form. The infinitive, inflected, countable noun and unrelated conditions were the same as in Experiment 1.

**Table 3:** Experiment 2: experimental conditions and examples of prime and target phrases.

	Condition	Prime phrase		Target phrase	
		Part 1	Part 2	Part 1	Part 2
1	Identical	das ‘the’	MIETEN ‘renting’	das ‘the’	MIETEN ‘renting’
2	Conversion.2	beim ‘at/during the’	MIETEN ‘rent’	das ‘the’	MIETEN ‘renting’
3	Inflected	wir ‘we’	MIETEN ‘rent’	das ‘the’	MIETEN ‘renting’
4	Infinitive	wir müssen ‘we have to	MIETEN ‘rent’	das ‘the’	MIETEN ‘renting’
5	Countable noun	mit den zwei ‘with the two’	MIETEN ‘rents’	das ‘the’	MIETEN ‘renting’
6	Unrelated	wir wollen ‘we want to’	WEINEN ‘cry’	das ‘the’	MIETEN ‘renting’

#### Results

**Figure 2:**
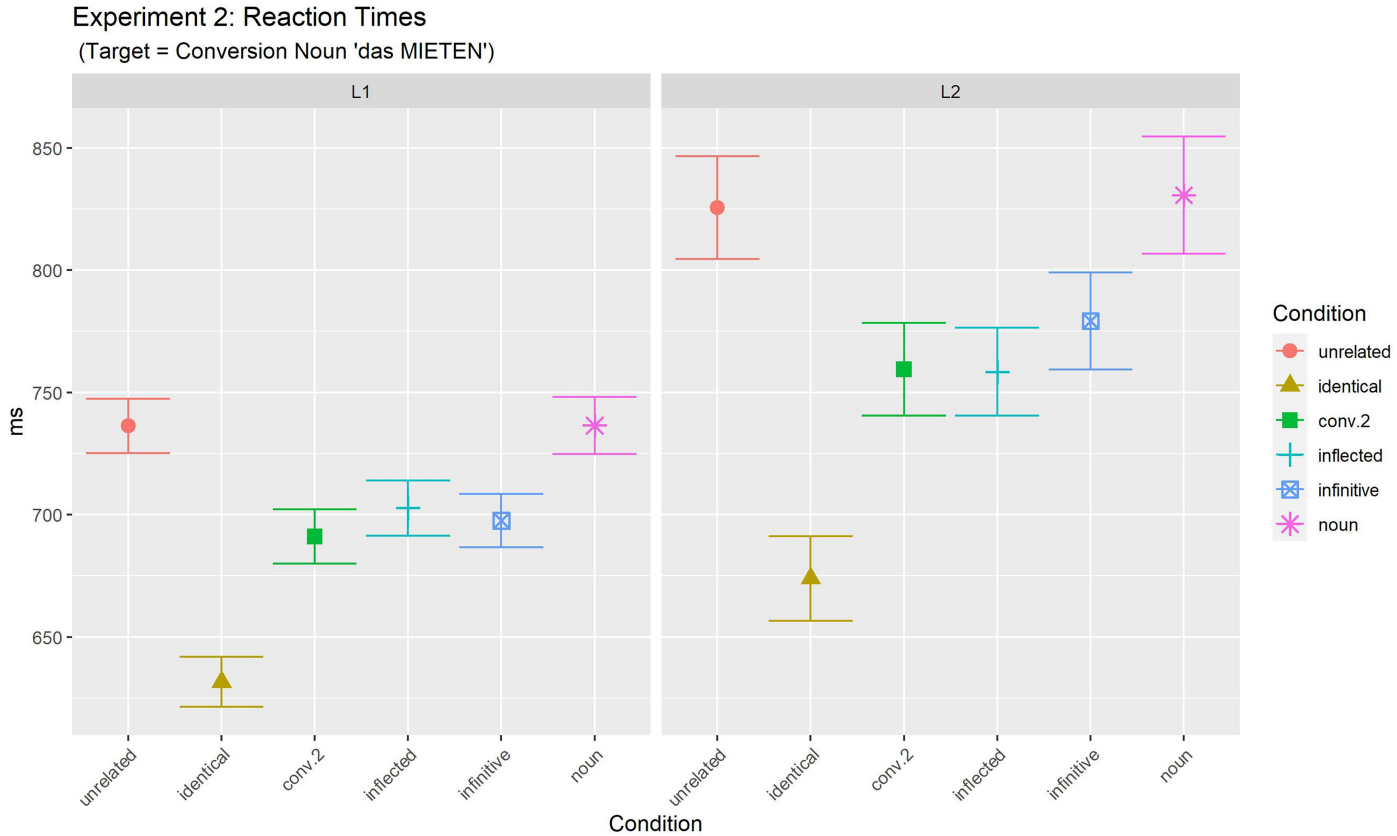
Experiment 2 (target: conversion noun): mean reaction times for target phrases.

Analyses were performed over the responses to which participants responded correctly both for the prime and target phrases, i.e., for the analyses of targets, we also pairwise excluded trials with incorrect responses to primes. This led to the exclusion of 13.8% (431 out of 3,112 total trials) of the data.^10^ Table 4 summarises mean latencies of the judgement task together with the number of observations for each cell (see also Figure 2).

**Table 4:** Experiment 2: mean reaction times for target phrases in ms, standard deviations (in brackets).

		Unrelated	Identical	Conv.2	Inflected	Infinitive	Noun	*Mean*
L1	RT (SD)	736.3 (181.1)	631.7 (164.1)	691.1 (180.8)	702.7 (184.5)	697.5 (177.2)	736.5 (182.4)	*699.3*
L2	RT (SD)	825.6 (284.7)	674.0 (228.3)	759.5 (270.2)	758.5 (260.7)	779.3 (289.1)	830.7 (280.5)	*771.3*
*Mean*		***780.9***	***652.9***	***725.3***	***730.6***	***738.4***	***783.6***	***735.3***

Statistical analyses [final model: log(RT) ~ Condition*Language + (1 | Participant) + (1 + Language | Item)] revealed a significant effect of Condition [*F*(5, 2506.2) = 35.64, *p <* 0.001], and an effect of Language [*F*(1, 129.5) =10.01, *p =* 0.002]. However, there was no significant interaction of the two factors [*F*(5, 2506.5) = 1.63, *p =* 0.149]. The main effect of Language indicates generally slower responses for L2 participants than for L1 participants.

In order to further investigate the main effect of Prime condition, differences between the levels of this factor were analysed by computing multiple comparisons of estimated means with adjusted values of *p* (Tukey). Details are summarised in Supplementary Material S1. Results of these comparisons reveal a clear pattern. Full priming was observed only for the identical (full repetition) condition. No priming was observed for the noun condition (770.5 ms) that was statistically identical with the unrelated (772.8 ms) condition (*p* = 1.00). The other three conditions (conversion.2, infinitive, inflected) all elicited partial priming. They were faster than the unrelated condition (all *p* < 0.001) and slower than the identical condition (all *p* < 0.001), but they were not different from each other (all *p* > 0.950), i.e., they all elicited partial priming of the same size. As indicated in Figure 2 and statistically substantiated by the non-significant interaction of Language:Condition, this pattern of priming results was the same for both populations (L1 and L2).

### Discussion of Experiments 1 and 2

Results revealed different priming patterns for conversion nouns and infinitives. Conversion nouns were partially primed by the inflected verb forms in Experiments 2, so that the priming between the two forms is symmetrical (partial priming was also reported for the reversed order, i.e., conversion nouns to inflected verbs, in Bordag and Opitz, 2021). However, infinitives were fully primed by the inflected forms in Experiment 1, which is in contrast to the results of Bordag and Opitz (2021), where infinitives only partially primed the target inflected forms. This priming pattern is the first indication that infinitives and conversion nouns differ in their representations, with inflected verbal forms being closer to infinitives than to conversion nouns. The priming asymmetries between the forms (bidirectional partial priming between conversion nouns and inflected forms, but asymmetric partial/full priming between infinitives and inflected forms) further suggest that the linguistic category of non-finites is not homogeneous/uniform in its mental representation and in particular in relation to other members of a word family. Moreover, the priming pattern between the inflected forms and infinitives is in accordance with the previous finding concerning adjective forms and verb stems (cf. for L1: Clahsen et al., 2001; Krause et al., 2015; Bosch and Clahsen, 2016; and also for L2 with early AoA: Bosch et al., 2019) that less specified forms (infinitives that do not express person and number) are less effective primes for more specific targets (inflected forms) while specific forms efficiently prime less specific forms.

In Experiment 1, we further observe that infinitive forms with ‘*um zu’* fully prime target infinitives with modal verbs. This finding is important among other things because it shows that priming in the experiments does not depend on repetition (or its absence) of the linguistic material in the first step of the presentation of primes and targets: The beginning of the phrases ‘*um zu*’ und ‘*wir müssen*’ is different in multiple respects and yet they induce full priming that obviously has its source in access to the same representation with the same function in prime and target.

Second, we observe yet another indication regarding different representations of infinitives and conversion nouns: While two different syntactic uses of infinitives fully prime infinitives as targets in Experiment 1, the situation is different for conversion nouns in Experiment 2: We do not observe full, but only partial priming between conversion nouns in a different syntactic use (i.e., in prepositional phrases with dative (*beim MIETEN*) vs. the conversion noun in the nominative phrase in targets (*das MIETEN*)). This finding suggests asymmetries between forms with different functions within one nominal paradigm. However, this conclusion is only speculative, since we do not have evidence that the form with nominative function would better prime the form in dative. Yet, the finding does contrast with the findings regarding the conjugated forms within a verbal paradigm that fully prime each other (see also Experiment 3 with target ‘*du gründest’*). In addition, the more specific dative form does not seem to be an effective prime for the nominative form that could be considered less specific, which is in contrast with previous findings in the adjective and verb domains.

The last important observation regarding these two experiments is that, in Experiment 1, there is a partial priming for derived countable nouns, i.e., these forms facilitate the recognition of the infinitive forms, though the facilitation is weaker than the partial priming in the conversion noun condition. This is surprising because derived countable forms facilitated neither inflected forms (in former studies, cf. Bordag and Opitz, 2021; Opitz and Bordag, 2021) nor conversion nouns in Experiment 2 of the present study. The finding suggests a special position of infinitive forms in morphological families that needs further exploration. It also indicates a different relationship between conversion nouns and infinitives, on the one hand, and countable derived nouns and infinitives, on the other. This asymmetry can be related to the productivity, regularity and semantic predictability of both forms: While conversion nouns are productive, regularly built and with predictable semantic relation to their base verb, derived countable nouns are not productive, irregularly built and both grammatically (e.g., gender) and semantically unpredictable. In addition, the countable derived nouns were presented in dative plural, which, as already mentioned, could have played a role, too (see section “General Discussion”).

## Experiments 3 and 4

In the following two experiments, we wanted to explore, in particular, the representation of conversion nouns in comparison with regularly derived nouns. The rationale of this step was that relationships between the morphological forms might be determined by their more specific properties such as semantic and grammatical predictability, or regularity of their formation rather than the adherence to a particular formation type (inflection/conversion vs. derivation). We thus included regularly derived nouns in the prime set of Experiment 3 and they were also targets in Experiment 4. To do so, we had to leave the strategy of employing the same forms with different functions in the experiments. In order to examine whether this change would result in changes of the priming pattern, we used a very similar design to the experiment in Bordag and Opitz (2021), but instead of presenting the inflected form *wir gründen* ‘we establish’ as a target, we used the form *du gründest* ‘you establish’, so that there is a non-identical relationship between the primes and targets in all conditions.

### Experiment 3

In this experiment, a target was always a finite verb form in the second person singular, e.g., *du gründest* ‘you establish’ which appeared also as a prime in the identical (full repetition) condition. The structure of the primes in the inflected, conversion and infinitive prime conditions was the same as in Experiments 1 and 2 and also in experiments in Bordag and Opitz (2021). The countable noun condition was replaced by a regular derivation condition which involved derivation by a very productive and regular affix (i.e., *-ung*). Thus, all derived forms in Experiment 3 were grammatically and semantically regular: They have a feminine gender and a meaning of abstractness. With these properties, the derived nouns are closer to conversion nouns than the countable derived nouns employed in previous experiments.

#### Methods

##### Participants

###### L1

All 70 participants were German native speakers (mean age = 26.4, SD = 6.3, range: [19, 45]; sex: 70.0% females, 28.6% males, 1.4% other), and most of them were students of different faculties at Leipzig University.

###### L2

A total of 66 non-native participants entered the final analysis. All were native Czech speakers (mean age = 25.8, SD = 5.6, range: [19, 44]; sex: 65.2% females, 34.8% males) and were from the same population pool as the participants in the previous experiment. Their L2 skills were also on B2/C1 level and were assessed in the same manner as in Experiment 1. None of the L1 or L2 participants participated in any other experiments of this study.

##### Materials

Twenty-four German verbs were selected that were of average or high frequency (ranged between frequency class 8 and 17 according to Leipzig Wortschatz Projekt) and well known to L2 learners at B2 level (as confirmed by a pretest). Items were chosen such that for each verb there is a derived noun that also falls into average to high frequency (frequency class range 8–15) and that is well known to the targeted L2 population. All nouns in the derived condition (see Table 5) were formed by a transparent (grammatically and semantically) affixation of *-ung* which is very productive, highly regular grammatically (derived noun always has feminine gender) and semantically (abstract meaning), e.g., *gründen* ‘to found’/‘to establish’—*Gründung* ‘foundation’ (a list of all items is given in Appendix A2). In addition, also the structures of some of the fillers were adopted in order to balance the use of derivational affixes (including ungrammatical phrases with nouns ending in -*ung*, and other derivational affixes). Table 5 gives an example of a complete set of conditions for one item.

**Table 5:** Experiment 3: experimental conditions and examples of prime and target phrases.

	Condition	Prime phrase		Target phrase	
		Part 1	Part 2	Part 1	Part 2
1	Identical	du ‘you’	GRÜNDEST ‘establish’	du ‘you’	GRÜNDEST ‘establish’
2	Inflected	wir ‘we’	GRÜNDEN ‘establish’	du ‘you’	GRÜNDEST ‘establish’
3	Infinitive	sie soll ‘she must’	GRÜNDEN ‘establish’	du ‘you’	GRÜNDEST ‘establish’
4	Derived	die ‘the’	GRÜNDUNG ‘establishment’	du ‘you’	GRÜNDEST ‘establish’
5	Conversion	das ‘the’	GRÜNDEN ‘establishing’	du ‘you’	GRÜNDEST ‘establish’
6	Unrelated	der ‘the’	DIEBSTAHL ‘theft’	du ‘you’	GRÜNDEST ‘establish’

#### Results

Target RTs were analysed only for responses to which participants responded correctly both for the prime and target phrases. This led to the exclusion of 10.2% (329 trials out of 3,217 total trials) of the data. Results are summarised in Table 6.

**Table 6:** Experiment 3: mean reaction times for target phrases in ms, standard deviations (in brackets).

		Unrelated	Identical	Inflected	Infinitive	Derived	Conversion	*Mean*
L1	RT (SD)	729.8 (174.1)	648.4 (180.0)	656.4 (188.6)	687.9 (181.5)	689.4 (178.9)	690.5 (182.6)	*683.7*
L2	RT (SD)	836.6 (259.4)	677.6 (212.5)	688.5 (214.4)	743.3 (221.7)	750.8 (223.9)	741.4 (251.1)	*739.7*
*Mean*		***783.2***	***663.0***	***672.5***	***715.6***	***720.1***	***716.0***	***711,7***

Statistical analyses [final model: log(RT) ~ Condition* Language + (1 | Participant) + (1 | Item)] revealed a significant effect of Condition [*F*(5, 2724.7) = 41.35, *p <* 0.001], and an effect of Language [*F*(1, 133.2) = 4.39, *p =* 0.038]. However, there was also a significant interaction of the two factors [*F*(5, 2723.9) = 2.66, *p =* 0.021].

In order to further investigate the observed interaction and to test whether the pattern of results was different in L1 and L2, results were further analysed for the two populations separately. Details of the final pairwise comparisons between conditions for L1 and L2 are summarised in Supplementary Material S1. In sum, they show the same pattern of differences for both populations. While full priming was observed in the inflected condition (same as identical condition, *p* = 0.979 in L1, *p* = 0.876 in L2), the other three conditions (infinitive, derived and conversion noun) exhibited partial priming (all differed from the full repetition condition and from the unrelated condition). Moreover, the group of conditions with partial priming was homogeneous in that they did not differ from each other (all *p* > =0.974), i.e., they led to partial priming of the same size.

**Figure 3:**
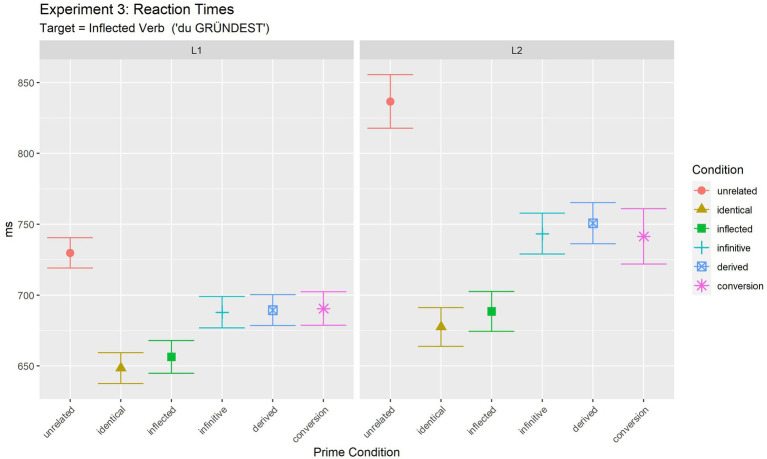
Experiment 3 (target: inflected verb): mean reaction times for target phrases.

Given this identical pattern in L1 and L2, the significant interaction observed in the main analysis thus indicates that the differences between conditions were more pronounced in L2 than in L1 (as also illustrated in Figure 3).^11^ Results are discussed in detail together with the results of Experiment 4.

### Experiment 4

The prime conditions in Experiment 4 were the same as in Experiment 3, but the targets were nouns regularly derived by a suffix -*er*. The function of this suffix is the same as in English and has the meaning of someone (or something) performing a particular action. All German nouns derived by this suffix have masculine gender.

#### Methods

##### Participants

###### L1

All 72 participants were German native speakers (mean age = 26.5, SD = 4.8, range: [19, 39]; sex: 66.7% females, 30.6% males, 2.8% other), and most of them were students of different faculties at Leipzig University.

###### L2

A total of 60 non-native participants entered the final analysis. All were native Czech speakers (mean age = 23.5, SD = 3.9, range: [19, 36]; sex: 70.0% females, 30.0% males) and were from the same population pool as the participants in the previous experiment. Their L2 skills were also on B2/C1 level and were assessed in the same manner as in Experiment 1. None of the L1 or L2 participants participated in any other experiments of this study.

##### Materials

The same 24 German verbs as in Experiment 3 were used. However, the target phrase this time always contained a nominal derivation of the verb with the affix -*er*. See Table 7 for an example of a full set of conditions for one item.

**Table 7:** Experiment 4: experimental conditions and examples of prime and target phrases.

	Condition	Prime phrase		Target phrase	
		Part 1	Part 2	Part 1	Part 2
1	Identical	ein ‘a’	GRÜNDER ‘establisher’	ein ‘a’	GRÜNDER ‘establisher’
2	Inflected	wir ‘we’	GRÜNDEN ‘establish’	ein ‘a’	GRÜNDER ‘establisher’
3	Infinitive	sie soll ‘she must’	GRÜNDEN ‘establish’	ein ‘a’	GRÜNDER ‘establisher’
4	Derived	die ‘the’	GRÜNDUNG ‘establishment’	ein ‘a’	GRÜNDER ‘establisher’
5	Conversion	das ‘the’	GRÜNDEN ‘establishing’	ein ‘a’	GRÜNDER ‘establisher’
6	Unrelated	er ‘he’	STIEHLT ‘steals’	ein ‘a’	GRÜNDER ‘establisher’

#### Results

**Figure 4:**
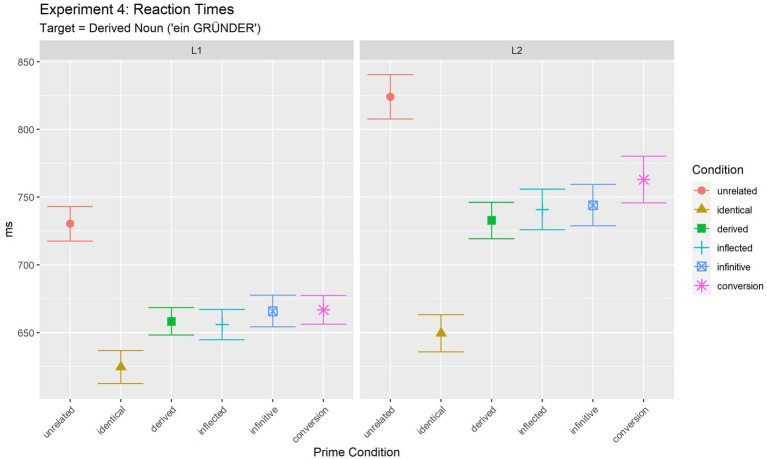
Experiment 4 (target: derived noun): mean reaction times for target phrases.

Target RTs were analysed only for responses to which participants responded correctly both for the prime and target phrases. This led to the exclusion of 9.32% (294 trials out of 3,145 total trials) of the data. Table 8; Figure 4 summarise mean latencies of the judgement task.

**Table 8:** Experiment 4: mean reaction times for target phrases in ms, standard deviations (in brackets).

		Unrelated	Identical	Derived	Inflected	Infinitive	Conversion	*Mean*
L1	RT (SD)	730.8 (204.2)	624.5 (203.0)	658.2 (167.9)	655.9 (181.2)	665.8 (195.9)	666.8 (160.1)	*666.7*
L2	RT (SD)	824.0 (237.7)	649.5 (205.0)	732.8 (202.3)	740.9 (227.4)	744.0 (228.3)	763.0 (219.0)	*742.4*
*Mean*		***777.4***	***637.0***	***695.5***	***698.4***	***704.9***	***714.9***	***704.7***

Statistical analyses [final model: log(RT) ~ Condition*Language + (1 | Participant) + (1 + Language | Item)] revealed a significant effect of Condition [*F*(5, 2678.4) = 46.88, *p <* 0.001], and an effect of Language [*F*(1, 127.0) = 12.66, *p =* 0.001] in addition to a significant interaction of the two factors [*F*(5, 2676.8) = 2.98, *p =* 0.011].

In order to further investigate the observed interaction and to test whether the pattern of results was different in L1 and L2, results were further analysed for the two populations separately. Results of the final pairwise comparisons between conditions for L1 and L2 are summarised in Supplementary Material S1. In sum, they show the same pattern of differences for both populations. All four conditions (derived, inflected, infinitive and conversion noun) differed both from the identical and the unrelated condition. All four conditions thus led to partial priming and did not differ from each other (all *p* > =0.513). This was observed in L1 and L2. In accordance with the visual impression given in Figure 4 (cf. Table 8), the significant interaction of the main analysis thus most likely indicates that the size of the partial priming was larger in L1 than in L2.^12^

### Discussion of Experiments 3 and 4

First, the results of Experiment 3 revealed that priming was not affected by primes and targets not being overtly fully identical compared to previous experiments. The inflected condition in this experiment (*GRÜNDEN*—*GRÜNDEST*) manifested full priming as the inflected condition with identical forms (*MIETEN*—*MIETEN*) did. Also, the results for the conversion and infinitive conditions were the same as in Bordag and Opitz (2021) and showed partial priming.

Further, the regularly derived -*ung* nouns led to partial priming of the same size as conversion nouns and infinitives. This contrasts with the absence of a priming effect for derived countable nouns in Experiment 2 and in Bordag and Opitz (2021). Moreover, in Experiment 4, we observe that a regularly derived noun (with suffix -*ung*) partially primes another regularly derived noun (with suffix -*er*). This contrasts with previous findings in English. Marslen-Wilson et al. (1994) observed no priming effects between two derived nouns (e.g., governor—government), while priming was observed between free stems (e.g., govern) and derived nouns (e.g., governor) in both directions. The authors explain the lack of priming effect by inhibiting links between competing derivational suffixes which cancel out the priming effects. Marslen-Wilson et al. (1996) suggested that this effect might be specific to auditory prime presentation (see also Giraudo and Grainger, 2001). However, in their unimodal (visual) priming experiments, no priming words derived by derivation suffixes -*lich* and -*heit* (in both directions) from the same stem were observed in Schriefers et al. (1992) in German either. Clearly, no evidence for such an inhibition mechanism can be observed in the German data presented here. In Experiment 4, derived nouns are partially primed to the same degree by inflected, infinitive and conversion nouns as well as by another derived noun. It should be noted that contrary to the derivational suffixes used by Schriefers et al. (1992) that have different word class marking (−*lich* adjective vs. -*heit* noun), both suffixes -*ung* and -*er* that were employed in the present study mark nouns. It could be thus the case that, at least in German, priming effects are more likely to appear between words from the same word class at least under some conditions (e.g., when they are derived).

In this context, we would like to mention Experiment 5 here on which we do not report fully. In this experiment, the target was again a deverbal noun derived by a suffix -*er* as in Experiment 4; this time from a verb stem with an inseparable prefix, e.g., *Befreier* (liberator). The deverbal noun primes had again the ending -*ung*, e.g., *Befreiung* (liberation). The inflected and conversion conditions were analogical to those in Experiment 4. The infinitive condition was replaced by a participle condition to include another non-finite form (e.g., *sie hat*—*BEFREIT*, ‘she has liberated’). The results exactly mirrored those of Experiment 4: Derived, inflected, conversion and participle conditions all partially primed the derived -*er* noun to the same degree. The results are thus replicable not only in the L1 and L2 comparison, but also with different linguistic material. Details for Experiment 5 are provided in the Supplementary Material.

## General Discussion

**Figure 5:**
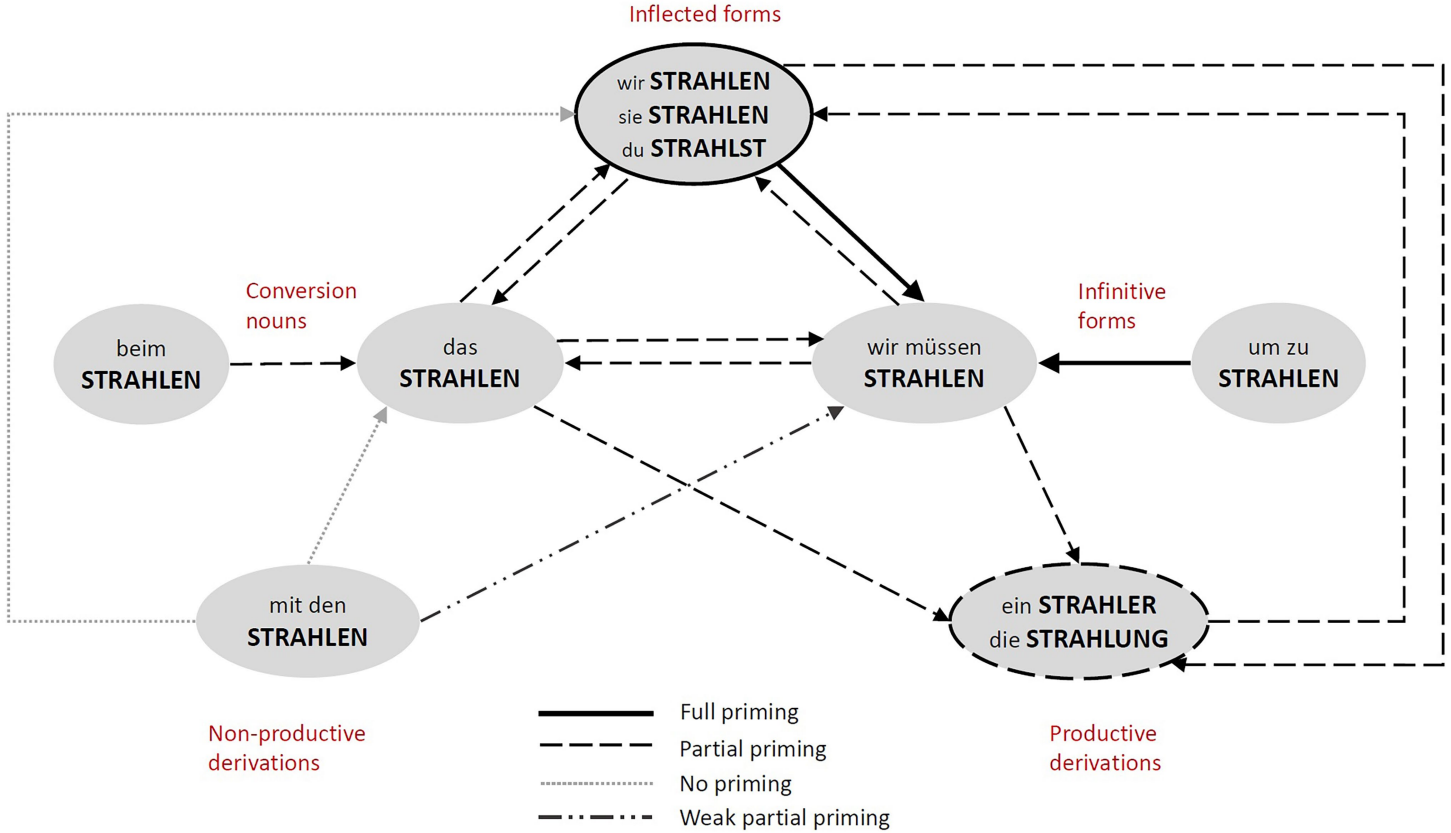
The figure represents a simplified summary of the priming relationships, focusing on the non-finite forms (conversion nouns and infinitives) in the centre. It combines the priming results of the current study and those of Bordag and Opitz (2021). The types of lines represent the different degree of priming between the forms. Arrows correspond to the priming direction. For the sake of clarity, some of the forms are collapsed into one circle (i.e., inflected forms and productive derivation) and the type of priming between them is represented in the line type of the circle (i.e., full priming between inflected forms, partial priming between productively derived forms).

The results of the experiments introduced in this study revealed several important findings about the psycholinguistic status of infinitives and conversion nouns and about the structure of representation of word families in German. They are summarised in Figure 5.

The examination of the directionality of priming between conversion nouns, infinitives and other members of the corresponding word families manifested a weaker relationship (partial priming) between these two forms than between finite forms within a verbal paradigm (full priming). This result indicates that neither infinitives nor conversion nouns have the same status as (other) inflected forms of the verbal paradigm, which also challenges the assumption of Haspelmath (1996) that conversion is another instance of inflection.

For conversion nouns, we did not find evidence that their status within the word family would be different from that of other productively and regularly derived nouns (partial priming). However, we found indications for a difference between the status of regularly and productively derived nouns (including conversion) and irregularly and unproductively derived countable nouns. These forms primed neither conversion nouns, nor finite forms, but they did partially prime infinitives in Experiments 1 in the present study. This partial priming was, however, weaker than the partial priming between the other forms in the experiment. This suggests that unproductive, irregular derivations are only weakly tied to other members of the word family. However, these derived forms in our experiment differed from the other derived forms not only in their productivity, but also because they were in dative plural. Results on priming within the conversion noun paradigm (Experiment 2) indicated that inflectional forms with more specific functions (dative singular) lead only to partial priming. Based on the available data, it is not possible to determine which of the two factors (productivity or the specific grammatical case), contributed to what extent to the marginal status within the word family that was seen for the unproductive derived forms in dative plural. Further research focussing on the issue is necessary to answer this question.

Infinitives, on the other hand, differed in their status from all other tested members of their word families. Similarly to the finite verb forms and contrary to all other tested word family members, they were fully primed by the finite verb forms. However, they themselves primed the finite verb forms only partially. Similarly, they also induced only partial priming for regularly derived nouns. Infinitives are thus closer tied to the finite verbal forms (asymmetric priming) than to the derived nouns (bidirectional partial priming) in the word family, but not as close as the finite forms between themselves (bidirectional full priming). The asymmetrical priming between the infinitives and the finite forms indicates a special status of infinitives that might be related to their underspecification as non-finite forms: When a finite verbal form is accessed, a verbal space comprising a rich set of verbal features is activated. As infinitive forms cover only a section of this space, they are fully primed when subsequently accessed. However, when an infinitive is accessed as a prime, only a section of the verbal space is activated and the target finite form thus receives only partial activation. Significantly, the two different infinitive types (bare infinitive and zu-infinitive) cover the same section of the verbal space and thus fully prime each other (however, only the direction with zu-infinitive as a prime was tested in Experiment 1). This argumentation parallels the interpretation of asymmetric priming reported between less and more specified stem forms in German (Clahsen et al., 2001; Krause et al., 2015; Bosch et al., 2019). The authors argue that the asymmetric priming in their studies originates in the different pairing of morphosyntactic features in the two conditions. In their more specified-to-less specified (*warf*- [+past] → *werf*-) condition, all information relevant for the target is already present in the prime. Therefore, the subsequent recognition of the target is more efficient in contrast to conditions when the prime does not include all features of the prime: In their less specified-to-more specified condition (*werf*- → *warf*- [+past]) the targets include additional information ([+past]), which results in significantly lower repetition priming.

This underspecification argument is more fitting than the overt frequency argument of, for example Schriefers et al. (1992), because it is not bound to the overt form, which was the same for (some of) the finite forms and the infinitive in our experiments. Moreover, this reasoning also agrees with current morphological frameworks that employ abstract feature decomposition and the concept of underspecification to explain inflectional patterns in paradigms and the distribution of different morphological exponents (e.g., Distributed Morphology; cf. Halle and Marantz, 1993, 1994), Paradigm Function Morphology (Stump, 2001) and Network Morphology (Corbett and Fraser, 1993). These underspecification-based frameworks are also supported by psycholinguistic evidence (e.g., Opitz et al., 2013, for sensitivity of ERP components for morphosyntactic specificity) and by evidence from impaired language in agrammatic aphasia (Janssen and Penke, 2002; Penke, 2009).

In sum, the asymmetric relationship between infinitives and inflected verb forms is in line with the assumption that words differ in their grammatical specificity (i.e., in the number of their grammatical features) and more complex specifications (i.e., more grammatical features) are more difficult to retrieve and process (Opitz et al., 2013), but they are better primes for other, less specific word forms (Clahsen et al., 2001; Krause et al., 2015; Bosch et al., 2019). In contrast, less specific items (i.e., comprising fewer or less specific grammatical features) tend to be faster and/or more easily retrieved and processed and better retained in agrammatic aphasics (Janssen and Penke, 2002), but they less effectively prime other, more specific forms. At the same time, partial priming between the dative form (more specific) and nominative form (less specific) of the conversion nouns in the present study is rather unexpected within the given framework but has been indicated also in previous research. For example VanWagenen and Pertsova (2014) found priming effects for a range of verbal inflectional affixes, but no significant effects for several nominal inflectional affixes in Russian. Differences in processing between various word classes are suggested also by Smolka and Ravid (2019). However, since our study was not designed to address such differences in specificity in the verbal vs. nominal domain, more research directly testing the (under)specification feature hypothesis within the noun paradigm and in direct comparison of other word classes is necessary to resolve the issue. In this research, various subgroups of nouns (e.g., underived nouns, regularly derived nouns, conversion nouns, etc.) should be included and compared to assess whether the differences between them could account for possibly differing (under)specification effects within the nominal paradigm. In addition, addressing the priming potential of different types of morpho-syntactic features that are associated with the verbs and nouns, in our experiments, could shed light on the issue, for instance, differences between interpretable (tense and aspect) and uninterpretable (agreement) features (see, e.g., Nanousi et al., 2006 on differences in production deficits in Greek-speaking agrammatic patients).

With respect to the classification of infinitives and conversion nouns in German, the forms do not manifest properties that would correspond to a psycholinguistically homogeneous class of non-finites. Infinitives seem to be more closely associated with verbal paradigms, while conversion nouns do not seem to differ from other derived nouns in a given word family as long as they are productively and regularly derived.

Apart from these issues that directly address German conversion nouns and inflected verbs within their word family, our results also offer insights into broader psycholinguistic issues such as discrete vs. gradient approaches to linguistic categories. Our results challenge the discrete category approach to complex word representations. In particular, neither inflection nor derivation seem to have homogeneous properties. As we have seen in the current study, the reliable partial priming of productively and regularly derived nouns indicates that these forms have a more central position in a word family than unproductive and irregular derived forms whose priming with other word family members is reduced or absent.

In the area of inflection, the inspected inflected verbal forms were very closely tied and mutually primed each other in both directions, irrespective of either full or partial form overlap; no asymmetries were observed. The situation seems to be different within nominal paradigms. As our study was not directed at this topic, we tested only priming between the dative singular forms of conversion nouns (*beim MIETEN*) and nominative singular forms (*das MIETEN*; that are overtly identical with accusative singular). However, despite the noun forms being overtly identical, the priming between them was only partial, which, as noted above, is not easily compatible with the (under)specification hypothesis. With respect to the priming behaviour of infinitives, they seem to linger between the verbal paradigm and derivations.

Some earlier studies suggest that transition between linguistic categories can be gradient from the psycholinguistic perspective or, put differently, that continua might better represent psycholinguistic relations than discrete categories. Within the area of derivation, previous studies show that processing of derived words may be affected by their lexical properties, such as the productivity of the formation/affix (Anshen and Aronoff, 1988, 1997; Vannest et al., 2005) or the semantic relation (transparent vs. opaque) between the base and the derivation in some languages (Smolka et al., 2019). Carota et al. (2016), who focussed on the role of semantic transparency and productivity in Italian, revealed that while transparent derivations with productive affixes are located at the one end of such continuum and opaque derivations with non-productive affixes at the other, the location of mixed derivations (e.g., opaque derivations with productive suffixes) depends on the degree of the affix productivity and semantic relatedness between the derivation and its base form. In the area of inflection, Smolka et al. (2013) explored whether linguistic categories are processed in continuous or categorical ways in the context of regular and irregular verbs (the distinction is directly related to productivity). Their results revealed that both behavioural and ERP priming effects were gradually affected by verb regularity, thus suggesting that the two linguistic categories are in fact processed continuously. Also, these findings indicate that linguistic categories are not homogeneous and that members within a single ‘category” may be subject to different morphological processes (e.g., morphological decomposition for the former and whole-word processing for the letter).

The so far inconclusive results regarding the processing of inflective vs. derived forms and the relationships between the representations within a word family can be due to the fact that the differences between the word family members and their processing do not simply go along the borderline between linguistic categories such as inflection and derivation, but rather represent a complex, possibly a language—or language-type-specific continuum characterised by multiple features/factors and comprising a transition area between its extremes where forms with mixed features are located. Under such a perspective, relations between all members of a word family may not be structured along categorical boundaries but in a more gradual manner (compare also to similar functionalist approaches to morphology, e.g., Bybee, 1985). Type of word formation as a scalar dimension has been proposed for example by Bertram et al. (2000), Kielar and Joanisse (2011), Smolka et al. (2013) and others. A gradient rather than a discrete approach to linguistic categories seems to be also better compatible with the presented data. Related to this problem is the question of how semantic transparency, which is unarguably of a gradient nature, is related to morphological relations in a word family and to productivity. Since our study was not designed to investigate this issue, the present data are not suitable for making strong statements about the role of semantic transparency, and we must refer to further research at this point.

One further issue that our present study contributes to concerns the role of context in language processing. Our specific experimental design, in which we manipulated the morphosyntactic context of prime and target words while keeping their form constant, not only minimizes form-related confounds for the investigated words, but also emphasizes the important role of the morphosyntactic context in retrieving (or activating) morphosyntactic features. While most previous morphological priming studies used isolated words, and morphosyntactic features were provided by means of the internal morphological structure of the words themselves (e.g., *visit-ed* vs. *visit-s*, *sing* vs. *sung*), the word forms in the present study (and thus their internal morphological structure and specifications) were ambiguous and only correctly interpretable by the information provided by the context.

Prominently, context-dependent processing and representation is often suggested in the area of semantics (e.g., in Distributed Semantics, starting conceptually with the distributional hypothesis by Firth, 1957; for recent review see, e.g., Boleda, 2020). However, also with respect to the processing and representation of morphosyntactic features, many theories attribute a prominent role to the context, formally either by means of syntactic structures that provide necessary morphosyntactic specifications for vocabulary insertion (e.g., Distributed Morphology, see Halle and Marantz, 1993, 1994; Harley and Noyer, 1999; Embick and Noyer, 2006), or, for instance, *via* the concept of constructions (e.g., in Construction Morphology, Booij, 2010, 2013). Although such theories differ in their notion of grammar and linguistic modelling quite substantially, they share the assumption that the interpretation of a word’s morphosyntactic specification crucially depends on the information of the context it appears in. This idea has long been supported also by psycholinguistic research (although sometimes without explicit references to such theories). With respect to morphosyntactic features, it has been demonstrated that some features inherent to words are not retrieved if their interpretation is not required by the context. For instance, when nouns are presented in isolation, their grammatical gender needs not be retrieved, as it is not required for grammatical processing and agreement marking (Schriefers, 1993; Levelt et al., 1999; Bordag et al., 2006). More recently, psycholinguistic evidence has been accrued suggesting that, for example phonetic/phonemic representations are dependent on the context they usually appear in and that determines their meaning or function (see, e.g., Gahl, 2008 on different representations of homophones, or Plag et al., 2017 on differences between morphemic and non-morphemic final /s/ and /z/ in English).

Our data thus support such accounts highlighting the role of context in language processing and lexical representations. The pattern of results observed in the present study could not easily be explained by accessing lexical representations without referring to the context they appear in. Our results thus emphasize the role of contextual information in morphosyntactic processing of words. They also shed doubts on the validity of results of experiments that investigated the priming of morpho-syntactic features by means of presenting isolated, single words without context. In the light of the present results, it is less clear which morphosyntactic feature representations (if any) are retrieved when encountering isolated words without context.

An anonymous reviewer suggested accounting for our data within a usage-based theory, such as Construction Grammar (e.g., construction morphology, see Booij, 2010, 2013). Though we acknowledge the role context plays in our study, it was not designed to test predictions of such theories and so we cannot present a conclusive response to this point. Although we agree that providing morpho-syntactic contexts in primes and targets, as in our study, is related to the idea of testing the priming of different constructions, we consider some aspects of the priming pattern in our data difficult to interpret within such approaches. As an example, we observe full or partial priming in cases, where the same morphological function appears, but no constructions are repeated (such as between *um zu SPIELEN* ‘in order to play’ vs. *wir müssen SPIELEN* ‘we have to play’). Moreover, since L2 learners are exposed to less input than L1 speakers, we would expect different or weaker priming patterns in L2 if these originated in constructions rather than in morphological relations between the investigated word forms. As already noted, the present study was designed to test other research questions. The method may, however, also inspire future research on the role of context in the processing of morphosyntactic features, possibly also within a usage-based framework.

The present study and its method may also inspire future research on the role of context in the processing of morphosyntactic features. For instance, a similar priming paradigm could be applied to investigate the priming potential of rather abstract morpho-syntactic configurations, i.e., addressing the question of abstract, item-independent morphosyntactic representations or constructions (e.g., in the sense of abstract paradigms, see Pinker, 2009). Moreover, theories on non-native processing would predict differences in the level of representation of such abstract configurations in L1 and L2 which would make such studies even more interesting (e.g., Shallow Structure Hypothesis by Clahsen and Felser, 2006, 2018, the Declarative-Procedural Model by Ullman, 2004, 2005).

In the present study, however, no differences in the priming patterns were observed between native German speakers and advanced German learners with L1 Czech. This result is congruent with previous findings in thematically related studies with the same populations and a similar experimental design (Bordag and Opitz, 2021; Opitz and Bordag, 2021). Despite this, the result is somewhat striking, since differences in L1 and L2 priming are often reported (see Jacob et al., 2018 for a review), though the same priming for L1 and L2 participants is sometimes observed as well (Feldman et al., 2010). Despite the mostly significant main effect for language revealing that the processing was more demanding for the L2 participants, their results manifested the same priming patterns as those of the L1 participants. This indicates that the L2 participants rely on the same representational structures and processing mechanisms as the L1 speakers with respect to the topics investigated in the present study. Several reasons might explain these results.

First, the L2 participants were advanced learners of German, and could thus be at a proficiency level, where the L2 representations and processing already closely approximate those of L1 (cf. Gor and Jackson, 2013, who found in an auditory priming experiment on Russian verbal inflection that American L2 learners of Russian show stronger priming effects approaching L1 patterns with increasing proficiency). However, differences in processing between these two populations have already been reported as well (Bordag et al., 2016, 2017; also Opitz et al., submitted).

Second, the relationship between the two languages could have played a role. Even though, for example the formal properties of the action nominals that surface as conversion nouns in German do not overlap with the formal properties of the corresponding Czech equivalents, the two language systems share some general properties that may result in similar organisation of word families in the mental lexicon. In particular, Czech is highly inflectional and German is one of the most inflecting languages in the Germanic language family. It could be the case that such similarities contribute to transfer of some general organising or structuring principles from L1 to L2 that then happen to coincide for both languages. Overall, we can say that the observed priming patterns were very coherent and robust and replicable in both L1 and L2 German.

Third, the fact that we obtained the same priming pattern for both L1 and advanced L2 speakers might be related to methodological issues. As explained in the introduction, our study relied on overt priming of word forms presented in context in order to guarantee adequate grammatical processing of the ambiguous word forms and ecological validity of the research. It could thus be the case that such an approach may ease the L2 processing and enable that it proceeds in a more native-like manner than when isolated word forms or even bound morphemes (see the irregular stem experiments of Krause et al., 2015; Jacob et al., 2018; Veríssimo et al., 2018) are presented which might be difficult to interpret and process especially for the L2 learners. In addition, Bosch and Clahsen (2016) observed a different priming pattern between L1 and L2 only when primes were masked. Similarly, Bosch et al. (2017) found that in the behavioural data, their L2 learners performed native-like, their ERP data, however, revealed L1/L2 differences with respect to the temporal dynamics of grammatical processing.

Clearly, more research is needed to understand the role of context in L1 and L2 processing and also to better understand the contributions of various methods. In addition, investigating non-native learners with either lower proficiencies or different temporal onsets of acquisition (cf. the AoA effects reported in Bosch et al., 2019) might help to further identify potential differences between L1 and L2. For the same purpose, future research may include learners with a typologically more different L1 or employ different research designs.

## Conclusion

To conclude, this study contributes to the understanding of how word families are organised in the mental lexicon. Our results on infinitives and deverbal conversion nouns in German, two linguistic forms whose morphological classification is still debated, together with additional empirical evidence reported in this study call for further research on these forms and on word family structure in general. More languages also need to be included in this line of research, since both experimental evidence (see Smolka and Ravid, 2019 and Milin et al., 2018 for overview) and computational models (Günther et al., 2019) indicate that quantitatively characterised differences between languages (e.g., degree of morphological analysis vs. synthesis) may result in behavioural observable differences in morphological representation and processing. Further research is also needed, for example to understand the relations between and within nominal and verbal paradigms and the relationship between morphologically complex words with different degrees of productivity and/or transparency. Methodologically, the study presented a paradigm that can open a new perspective on representation and morphological processing in at least two respects. On the one hand, it eliminates the problem of surface form overlap; on the other hand, and in particular, it takes into account syntactic context whose presence or absence in priming studies might have crucial impact especially on comparisons between native and non-native processing.

## Data Availability Statement

The datasets presented in this study can be found at the Open Science Framework (OSF): DOI 10.17605/OSF.IO/4FQ8K.

## Ethics Statement

The studies involving human participants were reviewed and approved by the German Research Council (DFG). The patients/participants provided their written informed consent to participate in this study.

## Author Contributions

All authors listed have made a substantial, direct and intellectual contribution to the work. The order of the authors’ names corresponds to the extent of their contribution.

## Funding

This work was supported by Deutsche Forschungsgemeinschaft (DFG, German Research Foundation; grant number BO 3615/6-2 to DB) and by Universität Leipzig within the program of Open Access Publishing.

## Conflict of Interest

The authors declare that the research was conducted in the absence of any commercial or financial relationships that could be construed as a potential conflict of interest.

## Publisher’s Note

All claims expressed in this article are solely those of the authors and do not necessarily represent those of their affiliated organizations, or those of the publisher, the editors and the reviewers. Any product that may be evaluated in this article, or claim that may be made by its manufacturer, is not guaranteed or endorsed by the publisher.

## Footnotes

^1^To our knowledge, masked priming has been used only with single words. Presenting longer phrases to prime syntactic structures would require longer processing time that would then exceed the usually very short time required for keeping the prime masked (i.e., at the threshold of conscious processing).

^2^As a reviewer pointed out, subtle differences in pronunciation of homophones have been observed in spoken language that could indicate non-overlapping form representations for homonymous words (Jurafsky et al., 2002; Gahl, 2008). Thus, the matter of formal overlap might be more complex and deserves a more fine-grained analysis, at least for the auditory modality. However, no such claims have been made for the written modality, where the formal input is clearly identical with a complete overlap for homonymous forms.

^3^A list with all items is provided in the Appendix A1.

^4^Frequency classes are based on the frequency of the most common word *der*—“the.” Frequency class of 12 means, e.g., that *der* occurs 2^12^ times as often as the words in this particular class (public access available *via* www.wortschatz.uni-leipzig.de).

^5^There would not be enough items fulfilling all requirements from only one of the groups.

^6^All critical forms were presented with capital letters to avoid orthographic cues to word categories, since in German spelling all nouns are written with a capital initial letter. Thus, the presented forms are more similar to the processing of the primary auditory mode, where no such additional cues are available.

^7^Randomisation was carried out with the program ‘mix’ (van Casteren and Davis, 2006).

^8^Since reducing the random effects structure in the final model by excluding random slopes for Condition increases the probability of Type 1 errors, we also computed maximum models that included all predictors (Condition and Language) as random slopes (for participants and items). Although none of the maximum models converged, we compared their results with those of the final models. These comparisons revealed essentially the same pattern of significant and non-significant effects for the tested main effects. We therefore conclude that the results of the final models can be viewed with a high degree of confidence.

^9^Raw data and R scripts together with detailed outputs of analyses are provided online (see Data Availability Statement).

^10^In this experiment, there was a larger number of incorrectly judged target phrases. This was especially so for the noun condition in which the conversion noun of the target was preceded by a countable noun in the prime. This can be explained by at least two factors: First, the conversion form of the targets in general are the most difficult forms, especially for L2 learners; second, for both groups of participants, the relatively higher error rates in the noun condition may be related to the difficulties of switching from one sense of a noun in the prime (*die zwei Mieten*, ‘the two rents’) to a related but different sense in the target noun (*das Mieten*, ‘the renting’). Or, from another perspective, processing of conversion nouns (here in targets) is much better prepared by all other, more closely related forms in contrast to the noun condition. However, it is important to note that although we do not discuss accuracy rates in detail here, they do not contradict the findings of reaction times. Both measures (RTs and accuracy) indicate more processing difficulties in the noun condition (longer RTs, more errors).

^11^Note that for the maximum (non-converging) model, this interaction was not significant, indicating that the significance of this interaction is potentially over-estimated in the final model (in contrast to the main effects of Condition and Language, for which also the maximum model yielded significant effects).

^12^Similar to Experiment 3, the interaction was not significant for the maximum (non-converging) model (in contrast to the significant main effects of Condition and Language).

## Appendix

**A1**. List of Items used in Experiments 1 and 2 (with English translations).

**Table:**

***Verb***		**Freq. class**	***Countable noun***	
*belegen*	to verify	10	*der Beleg*	the proof
*berichten*	to report	9	*der Bericht*	the report
*besuchen*	to visit	9	*der Besuch*	the visit
*beweisen*	to prove	10	*der Beweis*	the proof
*bremsen*	to brake	16	*die Bremse*	the brake
*bürsten*	to brush	12	*die Bürste*	the brush
*duschen*	to shower	12	*die Dusche*	the shower
*ernten*	to harvest	13	*die Ernte*	the harvest
*feiern*	to celebrate	13	*die Feier*	the celebration
*fischen*	to fish	17	*der Fisch*	the fish
*fliegen*	to fly	9	*die Fliege*	the fly
*löffeln*	to spoon	14	*der Löffel*	the spoon
*mauern*	to mason	13	*die Mauer*	the wall
*meistern*	to master	13	*der Meister*	the master
*mieten*	to rent	14	*die Miete*	the rent
*opfern*	to sacrifice	9	*das Opfer*	the sacrifice
*pflanzen*	to plant	8	*die Pflanze*	the plant
*schrauben*	to screw	16	*die Schraube*	the screw
*schulden*	to owe	14	*die Schuld*	the debt
*sorgen*	to worry	10	*die Sorge*	the worry
*speisen*	to dine	15	*die Speise*	the food
*teilen*	to share	14	*der Teil*	the part
*versuchen*	to try	10	*der Versuch*	the attempt
*zelten*	to camp	17	*das Zelt*	the tent

**A2**. List of Items used in Experiments 3 and 4 (with English translations).

**Table:**

***Verb***		***-ung derivation***		***-er derivation***	
*bohren*	to drill	*Bohrung*	*borehole*	*Bohrer*	*drill*
*dichten*	to write poetry	*Dichtung*	*poetry*	*Dichter*	*poet*
*erfinden*	to invent	*Erfindung*	*invention*	*Erfinder*	*inventor*
*erziehen*	to educate	*Erziehung*	*education*	*Erzieher*	*educator*
*färben*	to colour	*Färbung*	*color*	*Färber*	*dyer*
*fördern*	to promote	*Förderung*	*promotion*	*Förderer*	*promoter*
*forschen*	to research	*Forschung*	*research*	*Forscher*	*researcher*
*führen*	to lead	*Führung*	*leadership*	*Führer*	*leader*
*gründen*	to found	*Gründung*	*foundation*	*Gründer*	*founder*
*halten*	to hold	*Haltung*	*attitude*	*Halter*	*holder*
*heizen*	to heat	*Heizung*	*heating*	*Heizer*	*heater*
*kühlen*	to cool	*Kühlung*	*cooling*	*Kühler*	*cooler*
*lesen*	to read	*Lesung*	*reading*	*Leser*	*reader*
*ordnen*	to order	*Ordnung*	*order*	*Ordner*	*folder*
*prüfen*	to examine	*Prüfung*	*exam*	*Prüfer*	*examiner*
*rechnen*	to calculate	*Rechnung*	*invoice*	*Rechner*	*calculator*
*retten*	to save	*Rettung*	*rescue*	*Retter*	*savior*
*sammeln*	to collect	*Sammlung*	*collection*	*Sammler*	*collector*
*schalten*	to switch	*Schaltung*	*circuit*	*Schalter*	*switch*
*senden*	to send	*Sendung*	*broadcast*	*Sender*	*transmitter*
*strahlen*	to radiate	*Strahlung*	*radiation*	*Strahler*	*radiator*
*teilen*	to share	*Teilung*	*division*	*Teiler*	*divider*
*versichern*	to insure	*Versicherung*	*insurance*	*Versicherer*	*insurer*
*zeichnen*	to draw	*Zeichnung*	*drawing*	*Zeichner*	*drawer*
