# Supplementary material for: Mental Representation of Word Family Structure: The Case of German Infinitives, Conversion Nouns and Other Morphologically Related Forms
Source: Front Psychol. 2022 Jul 27;13:910849. doi: 10.3389/fpsyg.2022.910849 (PMC9363782; doi:10.3389/fpsyg.2022.910849)
Supplement: Supplementary file 1 [file Data_Sheet_1.PDF]

Supplementary Materials

to research article:
Opitz, A., Bordag, D. & Furgoni, A. (2022). Mental representation of word family structure: The case of German infinitives, conversion nouns and other morphologically related forms. Submitted to: *Frontiers in Psychology*.

Table of contents

S.1 Detailed Results for Pair-Wise Contrasts of Reaction Times on Targets **2**
- Experiment 1 2
- Experiment 2 3
- Experiment 3 4
- Experiment 4 5

S.2 Statistical Analyses of Accuracy **6**
- Experiment 1 6
- Experiment 2 7
- Experiment 3 9
- Experiment 4 9

S.3 Experiment S5 – Participle Forms **11**
- Participants 11
- Materials 11
- Results 12

S.3 Experiment S6 – Direction of Derivational Relation **15**
- Participants 15
- Materials 15
- Results & Discussion 16

S.1 Detailed Results for Pair-Wise Contrasts of Reaction Times on Targets

Experiment 1

Table S1. *Experiment 1: Reaction Times for Target Phrases: Estimated Means and Pairwise Contrast for Levels of Condition (Averaged over both Groups of Participants)*

**Contrast**	**estimate**	***SE***	**df**	**t-ratio**	**p**	***Signif.***
unrelated - identical	0.1746	0.0129	2783	13.56	<.001	***
unrelated - inf.zu	0.1529	0.0129	2783	11.88	<.001	***
unrelated - inflected	0.1560	0.0128	2783	12.15	<.001	***
unrelated - conversion	0.1087	0.0141	2792	7.72	<.001	***
unrelated - noun	0.0412	0.0131	2785	3.13	0.022	*
identical - inf.zu	0.0217	0.0129	2783	-1.69	0.541	
identical - inflected	0.0186	0.0128	2784	-1.45	0.696	
identical - conversion	0.0659	0.0141	2791	-4.68	<.001	***
identical - noun	0.1334	0.0131	2784	10.17	<.001	***
inf.zu – inflected	0.0031	0.0128	2784	0.24	0.999	
inf.zu - conversion	0.0442	0.0141	2792	-3.14	0.021	*
inf.zu - noun	0.1118	0.0131	2786	-8.52	<.001	***
inflected - conversion	0.0473	0.0140	2792	-3.37	0.010	**
inflected - noun	0.1148	0.0131	2786	-8.77	<.001	***
conversion - noun	0.0676	0.0143	2792	-4.72	<.001	***

Note. Degrees-of-freedom method: Kenward-Roger; *p* value adjustment: Tukey method for comparing a family of 6 estimates

Experiment 2

Table S2. *Experiment 2: Reaction Times for Target Phrases: Estimated Means and Pairwise Contrast for Levels of Condition (Averaged over both Groups of Participants)*

**Contrast**	**estimate**	***SE***	**df**	**t-ratio**	**p**	***Signif.***
unrelated - identical	0.1780	0.0153	2509	11.61	<.001	***
unrelated - conv.2	0.0869	0.0149	2511	5.81	<.001	***
unrelated - inflected	0.0783	0.0148	2507	5.28	<.001	***
unrelated - infinitive	0.0744	0.0149	2508	5.01	<.001	***
unrelated - noun	0.0012	0.0161	2510	-0.08	1.000	
identical - conv.2	0.0912	0.0151	2509	-6.02	<.001	***
identical - inflected	0.0997	0.0150	2511	-6.63	<.001	***
identical - infinitive	0.1036	0.0151	2512	-6.88	<.001	***
identical - noun	0.1792	0.0163	2510	-11.02	<.001	***
conv.2 - inflected	0.0086	0.0146	2508	-0.59	0.992	
conv.2 - infinitive	0.0125	0.0146	2505	-0.85	0.957	
conv.2 - noun	0.0881	0.0159	2513	-5.54	<.001	***
inflected - infinitive	0.0039	0.0145	2504	-0.27	0.999	
inflected - noun	0.0795	0.0158	2512	-5.03	<.001	***
infinitive - noun	0.0756	0.0158	2512	-4.78	<.001	***

Note. Degrees-of-freedom method: Kenward-Roger; *p* value adjustment: Tukey method for comparing a family of 6 estimates

Experiment 3

Table S3. *Experiment 3: Reaction Times for Target Phrases: Estimated Means and Pairwise Contrast for Levels of Condition, Separately for Language (Averaged over both Groups of Participants)*

L1

**Contrast**	**estimate**	***SE***	**df**	**t-ratio**	**p**	***Signif.***
unrelated - identical	0.1275	0.0177	2722	7.22	<.001	***
unrelated - inflected	0.1148	0.0177	2721	6.49	<.001	***
unrelated - infinitive	0.0630	0.0178	2721	3.55	0.005	**
unrelated – derived	0.0591	0.0177	2721	3.34	0.011	*
unrelated - conversion	0.0593	0.0183	2724	3.24	0.015	*
identical - inflected	-0.0126	0.0175	2721	-0.72	0.980	
identical - infinitive	-0.0644	0.0176	2721	-3.66	0.003	**
identical – derived	-0.0683	0.0175	2721	-3.90	0.001	**
identical - conversion	-0.0681	0.0182	2724	-3.76	0.002	**
inflected - infinitive	-0.0518	0.0176	2721	-2.94	0.039	*
inflected – derived	-0.0556	0.0176	2721	-3.17	0.019	*
inflected - conversion	-0.0555	0.0182	2723	-3.05	0.028	*
infinitive – derived	-0.0038	0.0176	2721	-0.22	0.999	
infinitive - conversion	-0.0036	0.0182	2724	-0.20	1.000	
derived – conversion	0.0001	0.0182	2723	0.01	1.000	

L2

**Contrast**	**estimate**	***SE***	**df**	**t-ratio**	**p**	***Signif.***
unrelated - identical	0.2152	0.0200	2727	10.763	<.001	***
unrelated - inflected	0.1943	0.0202	2728	9.640	<.001	***
unrelated – infinitive	0.1128	0.0201	2727	5.621	<.001	***
unrelated – derived	0.1098	0.0200	2728	5.486	<.001	***
unrelated - conversion	0.1258	0.0220	2730	5.712	<.001	***
identical - inflected	-0.0209	0.0188	2722	-1.114	0.876	
identical - infinitive	-0.1024	0.0187	2723	-5.467	<.001	***
identical – derived	-0.1053	0.0187	2723	-5.643	<.001	***
identical - conversion	-0.0893	0.0209	2732	-4.272	<.001	***
inflected - infinitive	-0.0814	0.0189	2722	-4.319	<.001	***
inflected – derived	-0.0844	0.0188	2722	-4.489	<.001	***
inflected - conversion	-0.0684	0.0211	2732	-3.250	0.015	*
infinitive – derived	-0.0029	0.0187	2722	-0.157	1.000	
infinitive - conversion	0.0130	0.0210	2730	0.621	0.989	
derived - conversion	0.0159	0.0209	2731	0.764	0.974	

Note. Degrees-of-freedom method: Kenward-Roger; *p* value adjustment: Tukey method for comparing a family of 6 estimates

Experiment 4

Table S4. *Experiment 4: Reaction Times for Target Phrases: Estimated Means and Pairwise Contrast for Levels of Condition, Separately for Language (Averaged over both Groups of Participants)*

**L1**						
**Contrast**	**estimate**	***SE***	**df**	**t-ratio**	**p**	***Signif.***
unrelated- identical	0.1600	0.0177	2669.5	9.02	<.001	***
unrelated - derived	0.0991	0.0178	2672.5	5.58	<.001	***
unrelated- inflected	0.1029	0.0180	2669.9	5.73	<.001	***
unrelated- infinitive	0.0905	0.0177	2669.9	5.12	<.001	***
unrelated- conversion	0.0837	0.0187	2675.7	4.46	0.001	***
identical - derived	-0.0609	0.0174	2667.9	-3.50	0.006	**
identical - inflected	-0.0571	0.0176	2667.6	-3.24	0.015	*
identical - infinitive	-0.0695	0.0173	2667.5	-4.01	<.001	***
identical- conversion	-0.0764	0.0184	2675.1	-4.14	<.001	***
derived - inflected	0.0038	0.0176	2667.9	0.22	0.999	
derived - infinitive	-0.0086	0.0173	2666.5	-0.49	0.996	
derived - conversion	-0.0154	0.0184	2676.4	-0.84	0.961	
inflected - infinitive	-0.0124	0.0176	2666.5	-0.71	0.981	
inflected - conversion	-0.0192	0.0186	2674.6	-1.03	0.907	
infinitive - conversion	-0.0069	0.0184	2673.2	-0.37	0.999	
**L2**						
**Contrast**	**estimate**	***SE***	**df**	**t-ratio**	**p**	***Signif.***
unrelated - identical	0.2409	0.0197	2677.0	12.26	<.001	***
unrelated - derived	0.1099	0.0196	2673.9	5.62	<.001	***
unrelated - inflected	0.1092	0.0195	2680.6	5.60	<.001	***
unrelated - infinitive	0.1049	0.0196	2679.1	5.34	<.001	***
unrelated - conversion	0.0733	0.0215	2689.4	3.41	0.009	**
identical - derived	-0.1309	0.0194	2681.6	-6.77	<.001	***
identical - inflected	-0.1317	0.0193	2677.0	-6.84	<.001	***
identical - infinitive	-0.1359	0.0194	2677.9	-7.01	<.001	***
identical - conversion	-0.1676	0.0213	2689.8	-7.87	<.001	***
derived - inflected	-0.0007	0.0192	2677.5	-0.04	0.999	
derived - infinitive	-0.0050	0.0193	2677.5	-0.26	0.999	
derived - conversion	-0.0366	0.0212	2691.1	-1.73	0.514	
inflected - infinitive	-0.0043	0.0192	2675.1	-0.22	0.999	
inflected - conversion	-0.0359	0.0211	2690.5	-1.70	0.532	
infinitive - conversion	-0.0316	0.0213	2690.3	-1.49	0.672	

Note. Degrees-of-freedom method: Kenward-Roger; *p* value adjustment: Tukey method for comparing a family of 6 estimates

S.2 Statistical Analyses of Accuracy

Experiment 1

Target phrases were analysed only if the corresponding prime phrase was judged correctly (exclusion of 264 data points out of 3264 data points, i.e.,7.5%). The overall accuracy rate for the remaining targets was very high (L1 = 99.1%; L2 = 98.8%98.9%). Mean accuracy rates for each of the conditions and both groups of participants are summarised in Table S5.

Table S5. *Experiment 1: Accuracy of Responses to Target Phrases in Percent*

	unrelated	identical	inf.zu	Inflected	conversion	noun	*mean*
L1	97.9	99.6	100.0	99.3	98.8	98.9	*99.1*
L2	98.8	98.8	99.2	99.6	100.0	97.0	*98.8*
mean	*98.4*	*99.2*	*99.6*	*99.5*	*99.4*	*98.0*	*98.9*

Evaluation of the results of a generalised linear mixed effect model (final model: Target.ACC ~ 1 + Condition + Language + Condition:Language + (1 | ParticipantID)) yielded no significant differences at all (all p>.254).

```
                   Chisq Df Pr(>Chisq)

Condition         6.5765  5     0.2541

Language          0.3547  1     0.5514

Condition:Language 3.4078  5     0.6374
```

In general, the very small differences in mean accuracy scores in the light of the very high overall accuracy rates do not allow for meaningful interpretation of possible differences between conditions (ceiling effect). However, they indicate that L1 & L2 participants were fully capable of dealing with the experimental task.

Experiment 2

Target phrases were analysed only if the corresponding prime phrase was judged correctly (exclusion of 206 data points out of 3112 data points, i.e., 6.6%). The overall accuracy rate for targets was high (91.8%). Mean accuracy rates for each of the conditions and both groups of participants are summarised in Table S6.

Table S6. *Experiment 2: Accuracy of Responses to Target Phrases in Percent*

	**unrelated**	**identical**	**conversion2**	**Inflected**	**infinitive**	**noun**	***mean***
L1	95.7	99.2	97.4	95.0	96.0	89.3	*95.4*
L2	88.5	92.1	92.3	93.8	93.4	67.8	*88.2*
mean	*92.1*	*95.7*	*94.9*	*94.4*	*94.7*	*78.6*	*91.8*

For most of the conditions, mean accuracy scores were very high. However, in the noun condition a strongly reduced accuracy rate was observed (89.3% in L1, 67.8% in L2). A generalised linear mixed effect model (final model:Accuracy ~ Condition + Language + Condition:Language + (1 | Participant) + (1 + Language | Item) confirmed the observation that more errors were made in the noun condition than in the other conditions (main effect for condition: $Chi^2(5)=100.3$, $p<.001$). Subsequent comparisons revealed significant lower accuracy rates in the noun condition compared to all other conditions (all $p<.001$). In addition, this effect was larger in L2 than in L1 (interaction Condition:Language: $Chi^2(5)=11.6$, $p=.041$).

Post-hoc pairwise contrasts:

```
Language = L1:
 contrast                estimate    SE  df z.ratio p.value
 unrelated - identical    -1.6454 0.785 Inf  -2.097  0.2887
 unrelated - conv.2       -0.4627 0.507 Inf  -0.912  0.9436
 unrelated - inflected     0.0765 0.431 Inf   0.177  1.0000
 unrelated - infinitive   -0.0302 0.456 Inf  -0.066  1.0000
 unrelated - noun          1.1022 0.384 Inf   2.871  0.0470
 identical - conv.2        1.1827 0.821 Inf   1.440  0.7023
 identical - inflected     1.7219 0.778 Inf   2.214  0.2312
 identical - infinitive    1.6152 0.789 Inf   2.046  0.3163
 identical - noun          2.7476 0.752 Inf   3.655  0.0035
 conv.2 - inflected        0.5391 0.496 Inf   1.087  0.8867
 conv.2 - infinitive       0.4325 0.515 Inf   0.840  0.9601
 conv.2 - noun             1.5649 0.456 Inf   3.432  0.0079
 inflected - infinitive   -0.1067 0.441 Inf  -0.242  0.9999
 inflected - noun          1.0258 0.370 Inf   2.773  0.0618
 infinitive - noun         1.1324 0.395 Inf   2.863  0.0481

Language = L2:
 contrast                estimate    SE  df z.ratio p.value
 unrelated - identical    -0.3242 0.370 Inf  -0.876  0.9522
 unrelated - conv.2       -0.4898 0.356 Inf  -1.377  0.7409
 unrelated - inflected    -0.7442 0.373 Inf  -1.996  0.3446
 unrelated - infinitive   -0.6991 0.365 Inf  -1.914  0.3935
 unrelated - noun          1.5406 0.294 Inf   5.234  <.0001
 identical - conv.2       -0.1656 0.392 Inf  -0.423  0.9983
 identical - inflected    -0.4200 0.409 Inf  -1.028  0.9087
 identical - infinitive   -0.3748 0.402 Inf  -0.932  0.9383
 identical - noun          1.8648 0.337 Inf   5.542  <.0001
 conv.2 - inflected       -0.2544 0.394 Inf  -0.645  0.9875
 conv.2 - infinitive      -0.2092 0.387 Inf  -0.541  0.9945
 conv.2 - noun             2.0304 0.323 Inf   6.286  <.0001
 inflected - infinitive    0.0451 0.403 Inf   0.112  1.0000
 inflected - noun          2.2848 0.343 Inf   6.669  <.0001
 infinitive - noun         2.2396 0.335 Inf   6.683  <.0001
```

Results are given on the log odds ratio (not the response) scale. P value adjustment: tukey method for comparing a family of 6 estimates

Results indicate that more errors were made in the noun condition and that this was more pronounced for the L2 participants.

Experiment 3

Target phrases were analysed only if the corresponding prime phrase was judged correctly (exclusion of 251 data points out of 3217 data points, i.e., 7.8%). The overall accuracy rate for remaining targets was high (97.4%). Mean accuracy rates for each of the conditions and both groups of participants are summarised in Table S7.

Table S7. *Experiment 3: Accuracy of Responses to Target Phrases in Percent*

	**unrelated**	**identical**	**inflected**	**infinitive**	**derived**	**conversion**	***mean***
L1	97.8	99.6	99.3	98.5	98.5	99.6	*98.9*
L2	94.0	98.4	93.6	95.6	96.4	95.3	*95.6*
mean	*95.9*	*99.0*	*96.5*	*97.1*	*97.5*	*97.5*	*97.4*

For most of the conditions, mean accuracy scores were very high and especially for L1 approaching ceiling. A generalised linear mixed effect model (final model:Accuracy ~ Condition + Language + Condition:Language + (1 | Participant) + (1 + Language | Item) confirmed the observation that more errors were made by L2 participants (main effect for Language: $Chi^2(1)=23.4$, $p<.001$). However, no significant influence of Condition was observed (no main effect: $Chi^2(5)=7.55$, $p=.183$); nor any interaction: $Chi^2(5)=3.77$, $p=.583$)).

Experiment 4

The overall accuracy rate for targets was high (97.4%). Mean accuracy rates for each of the conditions and both groups of participants are summarised in Table S4.

Table S8. *Experiment 4: Accuracy of Responses to Target Phrases in Percent*

	**unrelated**	**identical**	**derived**	**inflected**	**infinitive**	**conversion**	***mean***
L1	96.3	99.6	98.2	94.6	98.9	97.8	*97.6*
L2	94.6	99.6	97.0	99.1	96.1	95.9	*97.1*
mean	*95.5*	*99.6*	*97.6*	*96.9*	*97.5*	*96.9*	*97.4*

For most of the conditions, mean accuracy scores were very high and especially for L1 approaching ceiling. A generalised linear mixed effect model (final model:Accuracy ~ Condition + Language + Condition:Language + (1 | Participant) + (1 + Language | Item) indicated a significant interaction of Language:Condition ($Chi^2(5)=11.8$, $p=.038$). This interaction was further resolved by pairwise comparisons of conditions for L1 and L2 separately. Results revealed for no significant differences, neither for L1 nor for L2. Thus, the very small differences with very high overall accuracy rates do not allow for meaningful interpretation (ceiling effect). This was further confirmed by all non-significant posthoc pairwise comparisons.

Post-hoc pairwise contrasts:

Language = L1:

contrast	estimate	SE	df	z.ratio	p.value
unrelated - identical	-2.453	1.059	Inf	-2.315	0.1878
unrelated - derived	-0.813	0.567	Inf	-1.434	0.7059
unrelated - inflected	0.394	0.432	Inf	0.912	0.9436
unrelated - infinitive	-1.370	0.675	Inf	-2.029	0.3258
unrelated - conversion	-0.558	0.568	Inf	-0.983	0.9236
identical - derived	1.639	1.105	Inf	1.483	0.6752
identical - inflected	2.846	1.043	Inf	2.728	0.0698
identical - infinitive	1.082	1.164	Inf	0.930	0.9389
identical - conversion	1.894	1.107	Inf	1.712	0.5241
derived - inflected	1.207	0.536	Inf	2.252	0.2143
derived - infinitive	-0.557	0.745	Inf	-0.748	0.9758
derived - conversion	0.255	0.650	Inf	0.392	0.9988
inflected - infinitive	-1.764	0.650	Inf	-2.714	0.0724
inflected - conversion	-0.952	0.537	Inf	-1.774	0.4826
infinitive - conversion	0.812	0.747	Inf	1.087	0.8870

Language = L2:

contrast	estimate	SE	df	z.ratio	p.value
unrelated - identical	-2.550	1.052	Inf	-2.425	0.1476
unrelated - derived	-0.634	0.499	Inf	-1.270	0.8013
unrelated - inflected	-1.940	0.779	Inf	-2.490	0.1270
unrelated - infinitive	-0.352	0.466	Inf	-0.755	0.9748
unrelated - conversion	-0.245	0.507	Inf	-0.483	0.9968
identical - derived	1.917	1.079	Inf	1.776	0.4817
identical - inflected	0.611	1.234	Inf	0.495	0.9964
identical - infinitive	2.198	1.065	Inf	2.065	0.3060
identical - conversion	2.305	1.083	Inf	2.128	0.2725
derived - inflected	-1.306	0.815	Inf	-1.602	0.5975
derived - infinitive	0.281	0.525	Inf	0.536	0.9947
derived - conversion	0.389	0.563	Inf	0.691	0.9830
inflected - infinitive	1.588	0.796	Inf	1.994	0.3456
inflected - conversion	1.695	0.821	Inf	2.064	0.3062
infinitive - conversion	0.107	0.532	Inf	0.202	1.0000

Results are given on the log odds ratio (not the response) scale.
P value adjustment: tukey method for comparing a family of 6 estimates

S.3 Experiment S5 – Participle Forms

In this experiment, the infinitive condition was replaced by another non-finite form, i.e., by a participle condition (see Examples below).

Participants

A total of 65 participants were tested. They were all German native speakers (mean age = 27.9, SD = 6.2, range: [18, 42]; Sex: 69.2% females, 30.8% males).

Materials

30 verbs that formed participles regularly (through affixation of -*t*) but without prefixation were chosen as items. A subgroup of regular verbs in German form their past participle without (otherwise obligatory) additional prefixation of *ge*-. This subset of verbs is therefore more appropriate for the experimental design because they more closely resemble the forms of the other conditions. Because such verbs are usually less frequent, it cannot be assumed that all items are known to L2 speakers at the language levels studied (confirmed by pre-test). Therefore, this experiment was conducted only in L1.
A list of all items is given below in Table S13.

Table S9. *Example of items used in Experiment S5*

	**Condition**	**Prime Phrase**		**Target Phrase**	
		**Part 1**	**Part 2**	**Part 1**	**Part 2**
1	identical	ein<br>‘a’	BEFREIER<br>‘liberator’	ein<br>‘a’	BEFREIER<br>‘liberator’
2	inflected	wir<br>‘we’	BEFREIEN<br>‘liberate’	ein<br>‘a’	BEFREIER<br>‘liberator’
3	participle	er hat<br>‘he has’	BEFREIT<br>‘liberated’	ein<br>‘a’	BEFREIER<br>‘liberator’
4	derived	die<br>‘the’	BEFREIUNG<br>‘liberation’	ein<br>‘a’	BEFREIER<br>‘liberator’
5	conversion	das<br>‘the’	BEFREIEN<br>‘liberating’	ein<br>‘a’	BEFREIER<br>‘liberator’
6	unrelated	er<br>‘he’’	VERGISST<br>‘forgets’	ein<br>‘a’	BEFREIER<br>‘liberator’

Results

Accuracy

Table S10. *Experiment S5: Accuracy of Responses to Target Phrases in Percent*

	unrelated	identical	derived	inflected	infinitive	conversion	*mean*
L1	98.1	99.7	98.4	98.8	98.7	100	*98.9*

Accuracy was at ceiling level (98.9%). No differences between conditions were observed ($Chi^2(5)=2.83$, $p=.726$; final model: Accuracy ~ 1 + Condition + (1 | ItemID) + (1 | ParticipantID))).

Reaction Times

Table 11. *Experiment S5: Mean Reaction Times for Target Phrases in ms, Standard Deviations (in Brackets), and Number of Observations [in Square Brackets]*

	unrelated	identical	derived	inflected	participle	conversion	*mean*
**RT (SD)**	731.1 (189.9)	651.7 (175.8)	678.7 (185.4)	693.2 (206.0)	690.1 (198.1)	698.3 (175.5)	*690.4*

Figure S1. *Experiment S5 (Target: Conversion Noun): Mean Reaction Times for Target Phrases*

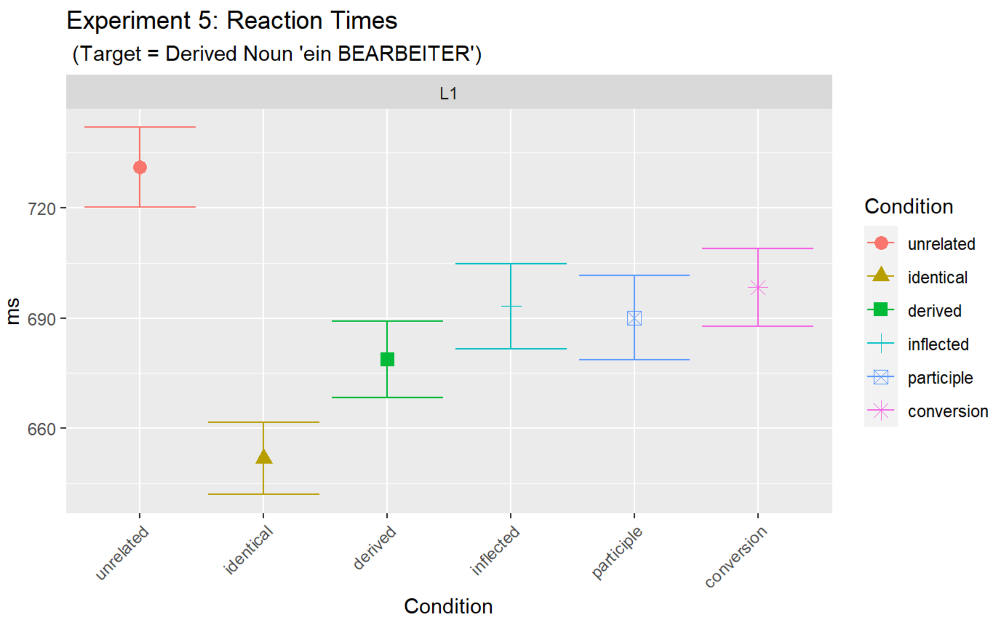

Analysis (final model: log(RT) ~ 1 + Condition + (1 | ParticipantID) + (1 | ItemID)) revealed a significant effect of Condition ($F(5, 1732.9) = 13.32$, $p < .001$). Subsequent tests showed that all four conditions (derived, inflected, participle, conversion noun) scored in between - and were significantly different from - the unrelated and identical condition. They all thus showed partial priming. The four conditions did not differ from each other (all p>=617).

Table S12. *Experiment S5: Reaction Times for Target Phrases: Estimated Means and Pairwise Contrast for Levels of Condition, Separately for Language (Averaged over both Groups of Participants)*

**Contrast**	**estimate**	***SE***	**df**	**t-ratio**	**p**	***Signif.***
unrelated - identical	0.1161	0.0145	1733	7.995	<.0001	***
unrelated - derived	0.0742	0.0146	1732	5.089	<.0001	***
unrelated - inflected	0.0642	0.0145	1732	4.416	0.0002	***
unrelated - participle	0.0634	0.0148	1733	4.284	0.0003	***
unrelated - conversion	0.0507	0.0151	1734	3.362	0.0102	*
identical - derived	-0.0419	0.0144	1731	-2.914	0.0420	*
identical - inflected	-0.0519	0.0144	1732	-3.614	0.0042	**
identical - participle	-0.0527	0.0146	1733	-3.602	0.0044	**
identical - conversion	-0.0654	0.0149	1734	-4.393	0.0002	***
derived - inflected	-0.0099	0.0144	1732	-0.691	0.9830	
derived - participle	-0.0107	0.0147	1734	-0.732	0.9780	
derived - conversion	-0.0234	0.0150	1733	-1.572	0.6174	
inflected - participle	-0.0007	0.0147	1733	-0.054	1.0000	
inflected - conversion	-0.0135	0.0149	1734	-0.907	0.9448	
participle - conversion	-0.0127	0.0152	1734	-0.840	0.9599	

Table S13. *List of items for Experiment S5*

Verb		***-ung derivation***		***-er derivation***	
übersetzen	to translate	*Übersetzung*	translation	*Übersetzer*	translator
benutzen	to use	*Benutzung*	usage	*Benutzer*	user
begründen	to give reasons	*Begründung*	reason	*Begründer*	founder
erobern	to conquer	*Eroberung*	conquest	*Eroberer*	conqueror
befreien	to liberate	*Befreiung*	liberation	*Befreier*	liberator
beobachten	to observe	*Beobachtung*	observation	*Beobachter*	observer
bearbeiten	to edit	*Bearbeitung*	editing	*Bearbeiter*	editor
erzählen	to tell	*Erzählung*	story	*Erzähler*	narrator
erforschen	to research	*Erforschung*	research	*Erforscher*	researcher
betreuen	to care	*Betreuung*	care	*Betreuer*	carer
entführen	to kidnap	*Entführung*	kidnapping	*Entführer*	kidnapper
vermieten	to rent	*Vermietung*	rental	*Vermieter*	landlord
verfassen	to write	*Verfassung*	constitution	*Verfasser*	writer
verfolgen	to pursue	*Verfolgung*	pursuit	*Verfolger*	pursuer
ermitteln	to investigate	*Ermittlung*	investigation	*Ermittler*	investigator
entwickeln	to develop	*Entwicklung*	development	*Entwickler*	developer
zerstören	to destroy	*Zerstörung*	destruction	*Zerstörer*	destroyer
bestellen	to order	*Bestellung*	order	*Besteller*	customer
erpressen	to blackmail	*Erpressung*	blackmail	*Erpresser*	blackmailer
unterdrücken	to oppress	*Unterdrückung*	oppression	*Unterdrücker*	oppressor
bewachen	to guard	*Bewachung*	guard	*Bewacher*	guard
begleiten	to accompany	*Begleitung*	company	*Begleiter*	companion
bekehren	to convert	*Bekehrung*	conversion	*Bekehrer*	proselytizer
bewundern	to admire	*Bewunderung*	admiration	*Bewunderer*	admirer
veranstalten	to organize	*Veranstaltung*	event	*Veranstalter*	organizer
verteidigen	to defend	*Verteidigung*	defense	*Verteidiger*	defender
begutachten	to examine	*Begutachtung*	examination	*Begutachter*	examiner
verteilen	to distribute	*Verteilung*	distribution	*Verteiler*	distributor
befürworten	to support	*Befürwortung*	support	*Befürworter*	supporter
betrachten	to look at	*Betrachtung*	contemplation	*Betrachter*	observer

S.3 Experiment S6 – Direction of Derivational Relation

In this priming experiment, we investigated whether the direction of the derivational relation between a verb and a related countable noun influences processing of the two forms themselves or that of related forms such as conversion forms.

For some of the items that were used in the experiments in our study, it can be assumed that the countable noun is historically derived from a verb, such as, for example in *kaufen* (to purchase) – *der Kauf* (the purchase). For other verbs, however, the derivational relation might be reversed historically, and the countable noun is the source of the derived verb, as for example in *der Löffel* (the spoon) – *löffeln* (to eat with a spoon) or *das Zelt* (the tent) – *zelten* (to camp). Please note, however, that it is notoriously difficult to determine the direction of derivation with certainty for many of such related word pairs (see, for example, Eisenheld 2021 for a discussion of directionality tests with zero-nouns in German).

As an anonymous reviewer of our study pointed out, if this type of directionality was reflected in the representation of verbs and corresponding countable nouns, potential influence of this factor on priming between them could not be excluded.. For instance, if a countable noun is the base of a verb as in *das Zelt* (‘tent’) – *zelten* (‘to camp’), this countable noun should better prime the infinitive (and inflected forms) of the verb as well as the conversion noun (*das Zelten* ‘the camping’) than it would be the case for the reversed derivational relation, for which additional derivational steps may be assumed.

In order to test whether this aspect plays a role in online processing, we conducted an experiment that targeted this particular question and that can be seen also as a control experiment for the current study on morphological families. This experiment was carried out only with native speakers in order to overcome limitations in item selection due to L2-proficiency levels. We also tested more items (36) in order to gain sufficient statistical power to detect potential effects. The design of this priming experiment was very similar to the experiments reported in the current paper, with the critical words in primes and targets embedded in minimal syntactic contexts that determined their meaning/grammatical function.

Participants

A total of 35 participants were tested. They were all German native speakers (mean age = 27.8, SD = 7.0, range: [19, 45]; Sex: 25 females, 10 males).

Materials

Thirty-six German verbs were selected. Half of them (18) were considered as having a derivational relation of noun → verb (*das Zelt – zelten.* ‘tent – to camp’), for half of them the assumed directionality of derivation was reversed (*kaufen – der Kauf*, ‘to purchase – the purchase’).

Derivational directionality was determined in a pre-test for which 50 potential item-candidates were selected and three experts (linguists) rated them as either belonging to one or the other group or as being ambiguous. Only words that all three raters independently rated as belonging to either group unambiguously were included in the experiment. A list of all items is given below in Table S16.

Similar to the experiments reported in our paper, all items were presented as primes and targets embedded in minimal phrases that determined their grammatical function/meaning. This time, however, all items were presented as either, a countable noun (N), an inflected verb (V), or a conversion noun (C), and these three forms were completely crossed for primes and targets yielding six different conditions. This way, the processing of verbs, nouns, and conversion forms as targets could be investigated when they were primed by each of the other forms/functions. Together with the factor of derivational direction, a total of 12 different conditions resulted, as is illustrated in Table S14 below.

Table S14. *Examples for experimental conditions in Experiment S6 (Zelt/zelten 'tent/to camp'; kaufen/Kauf 'to buy/the purchase'),*

		**Derivational Relation**			
		**N→V**		**V→N**	
**Condition**		**Prime Phrase**	**Target Phrase**	**Prime Phrase**	**Target Phrase**
1	C_V	das ZELTEN	wir ZELTEN	das KAUFEN	wir KAUFEN
2	N_V	das ZELT	wir ZELTEN	der KAUF	wir KAUFEN
3	C_N	das ZELTEN	das ZELT	das KAUFEN	der KAUF
4	V_N	wir ZELTEN	das ZELT	wir KAUFEN	der KAUF
5	N_C	das ZELT	das ZELTEN	der KAUF	das KAUFEN
6	V_C	wir ZELTEN	das ZELTEN	wir KAUFEN	das KAUFEN

Results & Discussion

Reaction times to target phrases are summarised in Table S15 and Figure S2 below. All procedures concerning data handling and statistical analyses were the same as reported in detail for the experiments in the main paper.

Table S15. *Experiment S6: Mean Reaction Times for Target Phrases in ms*

Condition	Derivational Relation	Mean RT	SD	N
C_V	N→V	586.1	159.7	194
C_V	V→N	561.7	146.6	198
N_V	N→V	610.2	148.3	206
N_V	V→N	588.5	135.5	202
C_N	N→V	623.2	145.7	186
C_N	V→N	611.1	152.0	202
V_N	N→V	608.0	142.0	201
V_N	V→N	620.7	151.3	200
N_C	N→V	676.1	176.2	188
N_C	V→N	660.0	165.6	194
V_C	N→V	632.0	172.2	193
V_C	V→N	632.3	164.0	200

Figure S2. *Experiment S6 (Direction of Derivational Relation): Mean Reaction Times for Target Phrases*

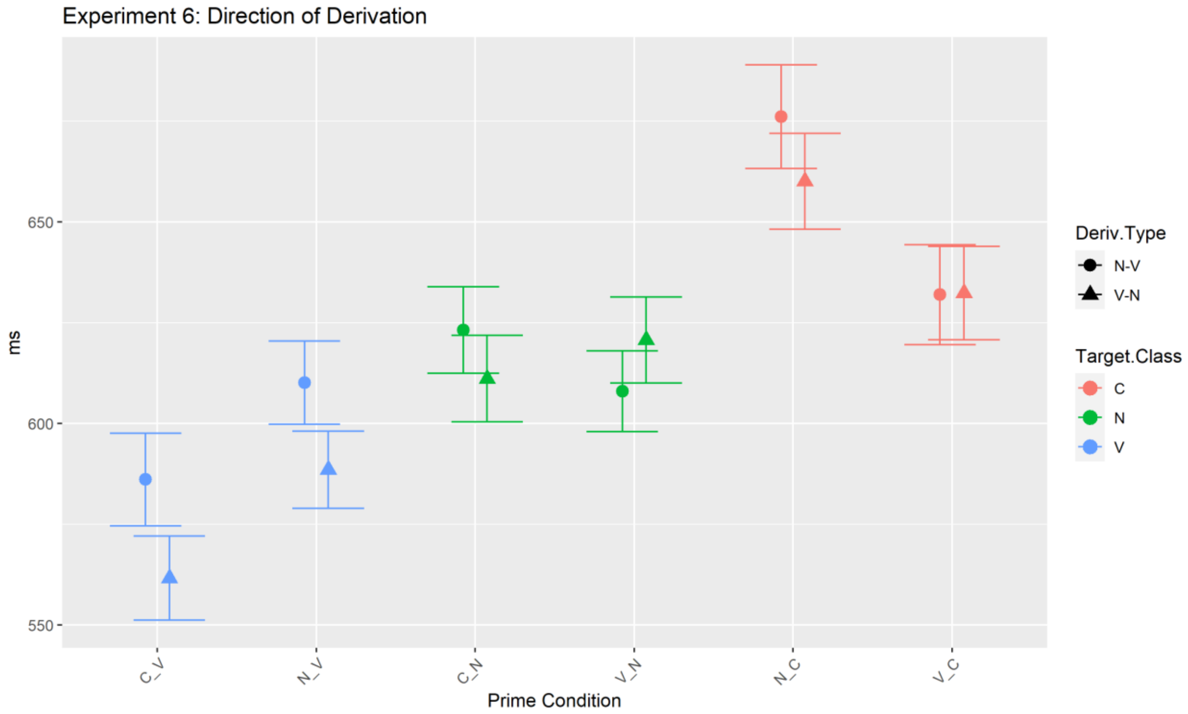

The final model (logRT ~ Condition*DerivationRelation + (1|Participant) + (1|Item)) revealed a significant main effect of condition (F(5, 2285.9) = 24.89, p< .001). The factor Derivational

Relation, however, failed to be significant (F(1, 33.9) = 1.92, p< .174). There was also no significant interaction of both factors (F(5, 2285.9) = 1.23, p< .293).

Subsequent analyses (pair-wise comparisons) of the significant main effect of condition revealed that fastest responses were seen for target verb forms that were preceded by a conversion noun (e.g., *das ZELTEN – wir ZELTEN*; *das KAUFEN – wir KAUFEN*), while the slowest responses were seen for conversion forms that were preceded by nouns as primes (*das ZELT – das ZELTEN*; *der KAUF – das KAUFEN*).

Results thus agree with the results reported in the main paper in that verbs were better primed by conversion nouns than by countable nouns (see blue conditions in Figure S2, i.e., C_V vs. N_V; see similar result in Bordag & Opitz 2021), and that conversion nouns were primed better by inflected verbs than by countable nouns (see red conditions in Figure S2, i.e., N_C vs. V_C; see similar result in Experiment 2). Also, similar to the results of Experiment 4 in the main paper (and Experiment 5 in supplementary), countable nouns were primed to a similar extend by inflected verbs and conversion forms (see green conditions in Figure S2, i.e., C_N vs. V_N).

Importantly, statistical analyses revealed no evidence that direction of derivation between verbs and countable nouns influenced priming of the targets. Therefore, we can assume that the directionality of derivation for the underlying verb – countable noun relation did not significantly affect the results reported in our main study either.

However, it should be noted that the lack of significant differences for the factor Derivational Relation does not exclude the possibility that this type of directionality is represented in the speakers' lexicon. Although we aimed at including prototypical examples of both directions as items and also increased statistical power by increasing the number of items, it could still be that the priming method is not sensitive enough to detect underlying representational and/or processing differences for this particular factor. As indicated in the Figure S2 above, especially for conditions with verbs as targets, there seemed to be a trend for faster responses for V→N type of items, at least numerically – although this observation was not substantiated statistically (difference between V→N vs. N→V for C_V p=0.078; for N_V p=.166). Employing other research methods such as EEG could shed more light on the topic.

To summarise, since we found no evidence that directionality of the derivational relation would influence participants' reaction to target phrases, we can conclude that it most probably also did not affect the results reported in the main paper that employed the same method.

Table S16. *List of items for Experiment S6*

	Verb	Countable noun	Direction of derivation	English Translation
1	FISCHEN	FISCH	N-V	to fish – a fish
2	FILMEN	FILM	N-V	to film – a film
3	KREISEN	KREIS	N-V	to circle – a circle
4	ZELTEN	ZELT	N-V	to camp – a tent
5	ÖLEN	ÖL	N-V	to oil – an oil
6	LÖFFELN	LÖFFEL	N-V	to spoon – a spoon
7	DUFTEN	DUFT	N-V	to smell – a smell
8	FORMEN	FORM	N-V	to shape – a shape
9	WÜRFELN	WÜRFEL	N-V	to throw dice – a die
10	BLITZEN	BLITZ	N-V	to flash – lightning
11	TIPPEN	TIPP	N-V	to guess – a hint
12	FÖHNEN	FÖHN	N-V	to blow-dry – a blow-dryer
13	RAUCHEN	RAUCH	N-V	to smoke – smoke
14	NERVEN	NERV	N-V	to go on somebody’s nerves – a nerve
15	TAGEN	TAG	N-V	to meet – a day
16	PFLANZEN	PFLANZE	N-V	to plant – a plant
17	GEIGEN	GEIGE	N-V	to play the violin – a violin
18	FLÖTEN	FLÖTE	N-V	to play the flute – a flute
19	TREFFEN	TREFF	V-N	to meet – a meeting
20	SITZEN	SITZ	V-N	to sit – a seat
21	GEWINNEN	GEWINN	V-N	to win – a prize
22	LOBEN	LOB	V-N	to praise – a praise
23	LEIDEN	LEID	V-N	to suffer – a sorrow
24	BERICHTEN	BERICHT	V-N	to report – a report
25	BAUEN	BAU	V-N	to build – a construction
26	ANTWORTEN	ANTWORT	V-N	to answer – an answer
27	SCHLAFEN	SCHLAF	V-N	to sleep – a sleep
28	TANZEN	TANZ	V-N	to dance – a dance
29	VERGLEICHEN	VERGLEICH	V-N	to compare – a comparison
30	KAUFEN	KAUF	V-N	to buy – a purchase
31	RUFEN	RUF	V-N	to call – a reputation
32	EMPFANGEN	EMPFANG	V-N	to receive – a reception
33	BEFEHLEN	BEFEHL	V-N	to order – an order
34	PFLEGEN	PFLEGE	V-N	to care – a care
35	DANKEN	DANK	V-N	to thank – gratitude
36	SIEGEN	SIEG	V-N	to win – a victory
